# Dysbiotic oral microbiota-derived kynurenine, induced by chronic restraint stress, promotes head and neck squamous cell carcinoma by enhancing CD8+ T cell exhaustion

**DOI:** 10.1136/gutjnl-2024-333479

**Published:** 2025-02-04

**Authors:** Fangzhi Lou, Li Yan, Shihong Luo, Yunmei Dong, Jingyi Xu, Ning Kang, Haiyang Wang, Yiyun Liu, Juncai Pu, Bing Yang, Richard D Cannon, Peng Xie, Ping Ji, Xin Jin

**Affiliations:** 1 College of Stomatology, Chongqing Medical University, Chongqing, China; 2 Chongqing Key Laboratory of Oral Diseases, Chongqing, China; 3 College of Medical Informatics, Chongqing Medical University, Chongqing, China; 4 NHC Key Laboratory of Diagnosis and Treatment on Brain Functional Diseases, The First Affiliated Hospital of Chongqing Medical University, Chongqing, China; 5 Department of Oral Sciences, Faculty of Dentistry, Sir John Walsh Research Institute, University of Otago, Dunedin, New Zealand

**Keywords:** CANCER, CARCINOGEN METABOLISM, EPITHELIAL BARRIER, IMMUNOREGULATION

## Abstract

**Background:**

Chronic restraint stress (CRS) is a tumour-promoting factor. However, the underlying mechanism is unknown.

**Objective:**

We aimed to investigate whether CRS promotes head and neck squamous cell carcinoma (HNSCC) by altering the oral microbiota and related metabolites and whether kynurenine (Kyn) promotes HNSCC by modulating CD8^+^ T cells.

**Design:**

4-nitroquinoline-1-oxide (4NQO)-treated mice were exposed to CRS. Germ-free mice treated with 4NQO received oral microbiota transplants from either CRS or control mouse donors. 16S rRNA gene sequencing and liquid chromatography-mass spectrometry were performed on mouse saliva, faecal and plasma samples to investigate alterations in their microbiota and metabolites. The effects of Kyn on HNSCC were studied using the 4NQO-induced HNSCC mouse model.

**Results:**

Mice subjected to CRS demonstrated a higher incidence of HNSCC and oral microbial dysbiosis than CRS-free control mice. *Pseudomonas* and *Veillonella* species were enriched while certain oral bacteria, including *Corynebacterium* and *Staphylococcus* species, were depleted with CRS exposure. Furthermore, CRS-altered oral microbiota promoted HNSCC formation, caused oral and gut barrier dysfunction, and induced a host metabolome shift with increased plasma Kyn in germ-free mice exposed to 4NQO treatment. Under stress conditions, we also found that Kyn activated aryl hydrocarbon receptor (AhR) nuclear translocation and deubiquitination in tumour-reactive CD8^+^ T cells, thereby promoting HNSCC tumourigenesis.

**Conclusion:**

CRS-induced oral microbiota dysbiosis plays a protumourigenic role in HNSCC and can influence host metabolism. Mechanistically, under stress conditions, Kyn promotes CD8^+^ T cell exhaustion and HNSCC tumourigenesis through stabilising AhR by its deubiquitination.

**WHAT IS ALREADY KNOWN ON THIS TOPIC**

- Oral microbiota and their metabolites are associated with higher risk of head and neck squamous cell carcinoma (HNSCC).
- Chronic restraint stress (CRS) is a tumour-promoting factor, but the mechanism of how it promotes HNSCC development remains unclear.

**WHAT THIS STUDY ADDS**

- CRS promotes HNSCC tumourigenesis and alters microbiota composition in 4-nitroquinoline-1-oxide- treated mice.
- Transplantation of saliva from CRS mice into germ-free mice results in dysbiotic oral microbiota, which may directly promote HNSCC tumourigenesis.
- The metabolomic profile is markedly altered by CRS-induced oral microbial dysbiosis, with an increase in the biosynthesis of kynurenine (Kyn).
- Under chronic stress conditions, Kyn promotes CD8^+^ T cell exhaustion and HNSCC tumourigenesis through stabilising aryl hydrocarbon receptor (AhR) by its deubiquitination.

**HOW THIS STUDY MIGHT AFFECT RESEARCH, PRACTICE OR POLICY**

- Our research indicates that CRS causes compositional alterations in microbiota and their metabolites, resulting in oral and gut barrier dysfunction, contributing to HNSCC tumourigenesis.
- The oral microbiota-Kyn-AhR pathway might serve as a novel therapeutic target for stress-induced HNSCC therapy.

## Introduction

 An estimated more than 460 000 new cases of head and neck squamous cell carcinoma (HNSCC) are diagnosed worldwide each year, with 5-year survival rates below 50%. HNSCC is highly invasive and frequently metastasizes to cervical lymph nodes, resulting in relapse and mortality.^1 2^ Apart from inherited genetic factors, smoking, alcohol consumption and human papillomavirus infection are environmental risk factors. Recent studies have found that chronic stress, as a psychobiological factor, significantly impacts the endocrine system, immune function and metabolic processes, playing a crucial role in the initiation and progression of tumours.^3 4^ Our previous studies using a well-established mouse model to induce chronic restraint stress (CRS), have shown that chronic stress enhances the tumour growth of OSCC in xenograft tumour assays.^5^ However, the detailed mechanism through which chronic stress promotes HNSCC initiation and progression remains unclear.

Increasing evidence suggests that the oral microbiota affects the immune response, metabolism and the occurrence and progression of cancer.^6 7^ A case–control study showed that the human oral microbiome is a prospective risk for pancreatic cancer.^8^ Recent studies provide supporting evidence that the oral microbiome is associated with risk for HNSCC,^1 9^ and oral microbiome dysbiosis promotes HNSCC progression.^10^ In addition, chronic stress can alter the composition and metabolic product secretion of the gut microbiota, influencing the immune system response and the progression of cancer.^11 12^ A recent epidemiological study found that the composition of the oral microbiota is associated with depression and anxiety symptoms in adolescents, suggesting that chronic stress may exert an influence on the oral microbial community.^13 14^ Nevertheless, whether the alteration of oral microbiota represents a connection between chronic stress and HNSCC remains unknown.

By superimposing CRS and HNSCC models in both specific pathogen-free (SPF) and germ-free (GF) mice, this study aims to investigate whether chronic stress promotes the progression of HNSCC by altering the oral microbiome and related metabolites, and if so, elucidate its molecular mechanisms.

## Materials and methods

### Animals

Female 5-week-old Kunming (KM) mice were purchased from the Cavensbiogle Model Animal Research Co. LTD (Jiangsu, China). These mice were maintained in the Animal Facilities of the Chongqing Key Laboratory of Oral Diseases under SPF conditions. Female 5-week-old GF KM mice were obtained from the NHC Key Laboratory of Diagnosis and Treatment of Brain Functional Diseases of the First Affiliated Hospital of Chongqing Medical University (Chongqing, China). The GF mice were kept in flexible film gnotobiotic isolators. All experiments for GF mice were performed in the NHC Key Laboratory of Diagnosis and Treatment of Brain Functional Diseases. No more than three mice were housed per cage.

### Statistical analysis

Data were expressed as mean±SD and were analysed by two-tailed Student’s t-test or one-way analysis of variance. Correlation analysis was performed using Pearson’s correlation coefficient. The incidence of tumour variables was assessed using Fisher’s exact test. The survival rates were evaluated by the log-rank test. To compare the consistency of microbiota changes induced by CRS exposure between SPF and GF mouse models, the fold change in abundance of each differential bacterium in the CRS group relative to the non-CRS group was calculated. Differences in bacterial abundance between sample groups were represented as log2-fold changes. Statistical analysis was conducted using GraphPad Prism V.9.0 software (La Jolla, USA) and R software (V.3.5.2). Differences were considered significant if p<0.05 and denoted by asterisks (*p<0.05, **p<0.01, ***p<0.001).

Additional methods are provided in online supplemental information.

## Results

### CRS promotes HNSCC tumourigenesis

**Figure 1:**
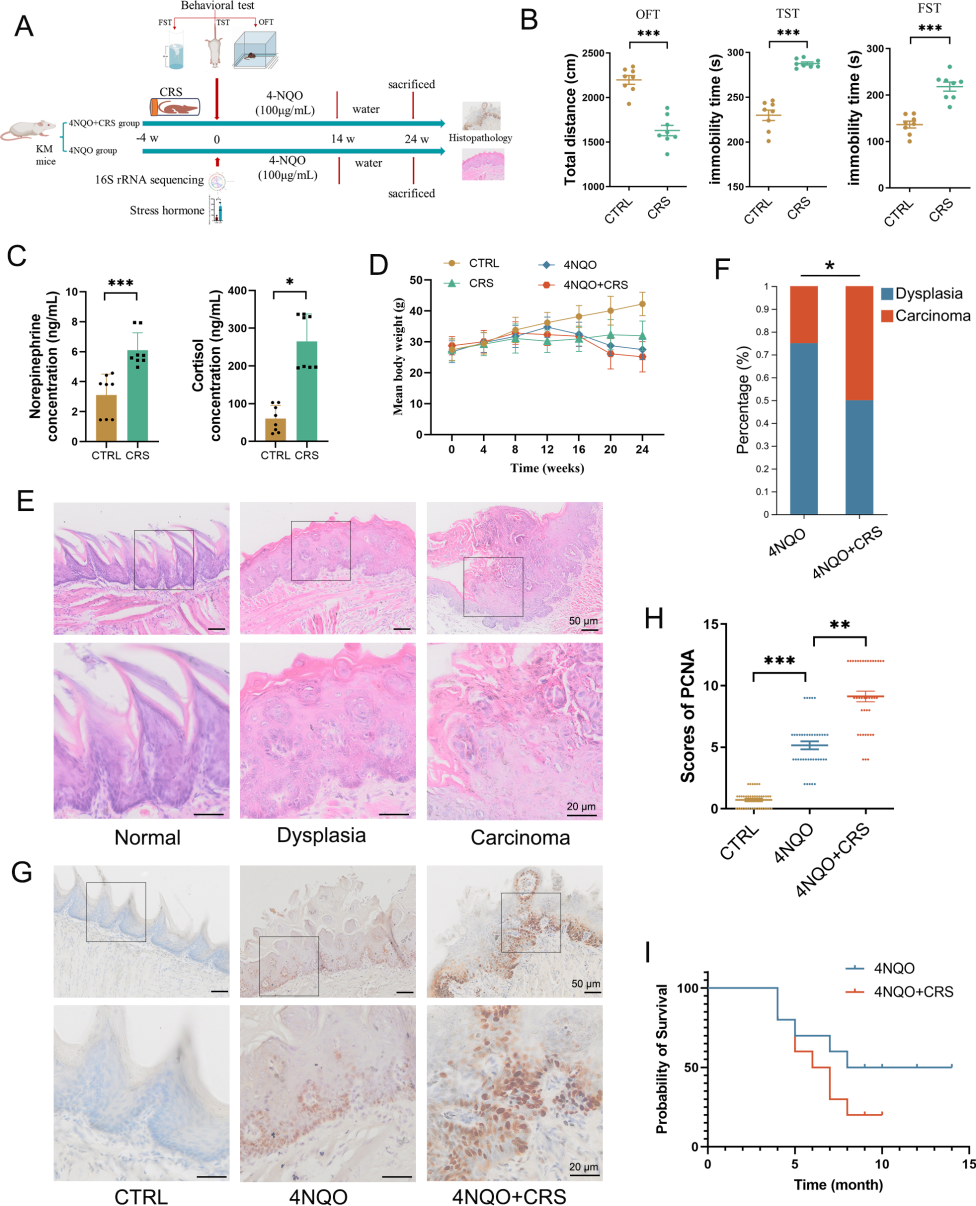
Chronic restraint stress (CRS) promotes HNSCC tumourigenesis in mice. (**A**) Schematic overview of the 4NQO-induced cancer model, with HNSCC induced under conditions of CRS or without CRS (4NQO group, n=8; 4NQO+CRS group, n=8). (**B**) The behavioural tests. The centre motion distance and immobility time of mice were measured after 4 weeks of CRS in the OFT, TST and FST using an EthoVision XT 13.0 system. (**C**) ELISA analysis measured the levels of NE and cortisol in mouse plasma. (**D**) Mean body weight of mice in the normal, CRS, 4NQO and 4NQO+CRS groups. (**E**) Representative images of H&E staining of normal epithelia, dysplastic epithelia and squamous cell carcinoma in mouse tongue tissue. (**F**) Prevalence of dysplasia and carcinoma in the tongue tissue of 4NQO-treated mice. Statistical significance was determined by Fisher’s exact test. (**G**) Representative images of immunohistochemcal staining of PCNA-positive cells in the tongue tissue. (**H**) The score for the number of PCNA-positive cells in the tongue tissue (n=5 fields from 8 mice). (**I**) Survival of 4NQO-treated mice. Data are expressed as mean±SD. Statistical significance was determined by two-tailed Student’s t-test. *p<0.05, **p<0.01, ***p<0.001. FST, forced swim test; HNSCC, head and neck squamous cell carcinoma; NE, norepinephrine; 4NQO, 4-nitroquinoline-1-oxide; OFT, open field test; PCNA, proliferating cell nuclear antigen; TST, tail suspension test.

To study the effect of chronic stress on HNSCC tumourigenesis in vivo, we exposed 4-nitroquinoline-1-oxide (4NQO)-treated mice to chronic restraint conditions (figure 1A). With 4 weeks of CRS, the centre motion distance measured with the open field test was significantly decreased and the immobility times in the TST and FST were significantly increased relative to the control (figure 1B), indicating anxiety-like and depression-like behaviour. ELISA of plasma samples showed that CRS stimulated the secretion of NE (a catecholamine hormone) and cortisol (a stress hormone) (figure 1C). The weight of mice in CRS group was substantially decreased compared with the control group (figure 1D). Histological staining of tongue tissue showed that the presence of dysplastic epithelia and squamous cell carcinoma and exposure to CRS significantly increased the incidence of tumours (figure 1 E,F). IHC staining showed that 4NQO induced the expression of the cell-proliferation protein marker proliferating cell nuclear antigen (PCNA), and the expression of PCNA was higher in the 4NQO+CRS group of mice than in the 4NQO group (figure 1 G,H). Moreover, exposure to CRS shortened the survival of mice significantly (figure 1I). These results demonstrate that CRS promotes 4NQO-induced HNSCC tumourigenesis in mice.

### CRS alters the oral microbiota composition

16S rRNA gene sequence analysis of saliva samples was performed to determine potential CRS-induced alteration of the oral microbiota. 3D-principal coordinate analysis (3D-PCoA) (beta diversity) based on Bray-Curtis distance revealed a distinct clustering of oral microbiota at the ASV level in mice in the control and CRS groups (online supplemental figure S1A). A Venn diagram also showed variation between the two groups, with the CRS group exhibiting 52 specific species of bacteria and the Control group exhibiting 31 specific species (online supplemental figure S1B). Community analysis confirmed the distinct oral microbial communities between the two groups (online supplemental figure S1C). Circos maps of communities showed the link between the samples and the species and effectively depicted the dominance of specific species within each group. The primary genera that dominated in the CRS group were *Pseudomonas* and *Veillonella* (online supplemental figure S1D). Oral bacteria including *Staphylococcus* and *Corynebacterium* genera were depleted with CRS exposure (online supplemental figure S1E). These results indicate that CRS alters the oral microbiota composition in mice.

### Transplantation of saliva from CRS mice into GF mice results in dysbiotic oral microbiota

**Figure 2:**
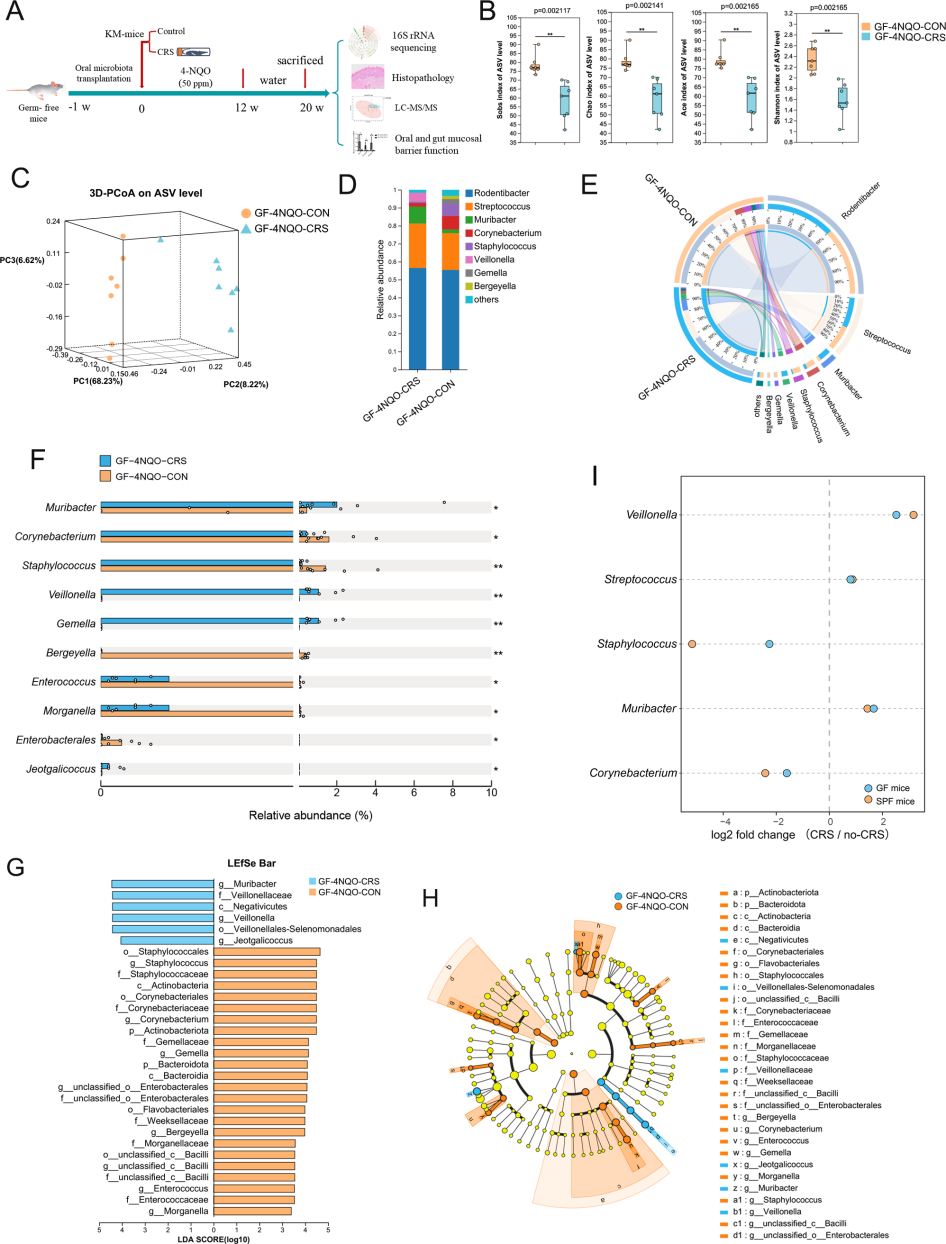
Saliva transplantation from CRS mice into GF mice results in dysbiotic oral microbiota composition. (**A**) A schematic overview of the transplantation of GF mice with oral microbiota from either CRS or control mouse donors (GF-4NQO-CON group, n=7; GF-4NQO-CRS group, n=7) that were then exposed to 4NQO treatment. (**B**) The alpha-diversity indices (including Sobs, Chao1, Ace and Shannon indices) of the GF-4NQO-CRS group and GF-4NQO-CON group. The significance of alpha diversity was assessed by a two-tailed Wilcoxon rank-sum test. (**C**) PCoA analysis (beta diversity) of the GF-4NQO-CRS group and GF-4NQO-CON group. The significance of beta diversity was assessed by ANOSIM. (**D**) The composition of the microbiota at the genus level in the GF-4NQO-CRS group and the GF-4NQO-CON group. (**E**) A Circos graph showing the distribution of microbial species at the genus level in the GF-4NQO-CRS group and the GF-4NQO-CON group. (**F**) Bacterial genera from oral swabs that had the greatest change in abundance between the GF-4NQO-CRS group and the GF-4NQO-CON group and their relative abundance. (**G**) LEfSe Bar chart identified the specific microbes with the top abundance that characterised each group. (**H**) Cladogram of the characterised microbes in each group, depicting their evolutionary and familial relationships. (**I**) Consistent alteration in bacteria abundance (p<0.05, CRS-exposed mice vs CRS-free mice; GF-CRS mice vs GF-CON mice) in two mice model (GF mice and conventional SPF mice). The fold change in abundance between CRS and non-CRS was calculated. *p<0.05, **p<0.01. CRS, chronic restraint stress; GF, germ-free; LEfSe, linear discriminant analysis effect size; NQO, 4-nitroquinoline-1-oxide; PCoA, principal coordinates analysis; SPF, specific pathogen-free.

To confirm the direct role of CRS-altered oral microbiota in HNSCC tumourigenesis, we transplanted oral microbes from CRS or control mouse donors to GF mice exposed to 4NQO treatment. The mice were euthanised and examined after 20 weeks (figure 2A). We performed 16S rRNA gene sequence analysis to explore the colonisation and composition of the oral microbiota after transplantation from CRS or control mice. Alpha diversity (including Sobs, Chao1, Ace and Shannon indices) was significantly decreased in GF mice transplanted with oral microbes from CRS-exposed mice (GF-4NQO-CRS) compared with the alpha diversity for GF mice transplanted with oral microbes from control mice (GF-4NQO-CON) (figure 2B). 3D-PCoA (beta diversity) based on Bray-Curtis distance revealed significant segregation of oral microbiota between these two groups of GF mice (GF-4NQO-CRS vs GF-4NQO-CON, p<0.05, ANOSIM) (figure 2C). Community analysis also confirmed the distinct oral microbial communities between the two groups (figure 2 D,E). Compared with the GF-4NQO-CON group, there were 10 bacterial genera the abundances of which were altered significantly (p<0.05, FC>1.5) in the GF- 4NQO-CRS group (figure 2F). Among these, the *Muribacter*, *Veillonella*, *Gemella* and *Jeotgalicoccus* genera were enriched while oral bacteria in the genera *Corynebacterium*, *Staphylococcus* and *Enterococcus* were depleted with CRS exposure (figure 2F). Linear discriminant analysis effect size (LEfSe) analysis showed that the GF-4NQO-CRS group was characterised by enriched ASVs belonging to the genera *Veillonella*, and *Jeotgalicoccus*, and the *Veillonellnceae* families compared with the GF-4NQO-CON group (figure 2 G,H). The consistence of microbiota alterations between the two mice models further was assessed. It was found that bacteria with increased abundance in CRS-exposed mice compared with control mice were consistently increased in GF-CRS mice compared with GF-CON mice, whereas bacteria with decreased abundance in CRS-exposed mice were consistently reduced in GF-CRS mice compared with GF-CON mice (figure 2I). Together, these results indicate that the composition of the oral microbiota in GF mice treated with 4NQO following oral microbiota transplantation from CRS-exposed mice was different to that in mice receiving microbiota from control mice. Moreover, oral microbiota transplantation in GF mice recapitulated the alterations of the oral microbiota observed in CRS-exposed conventional SPF mice.

### CRS-altered oral microbiota increases HNSCC tumourigenesis in GF mice

**Figure 3:**
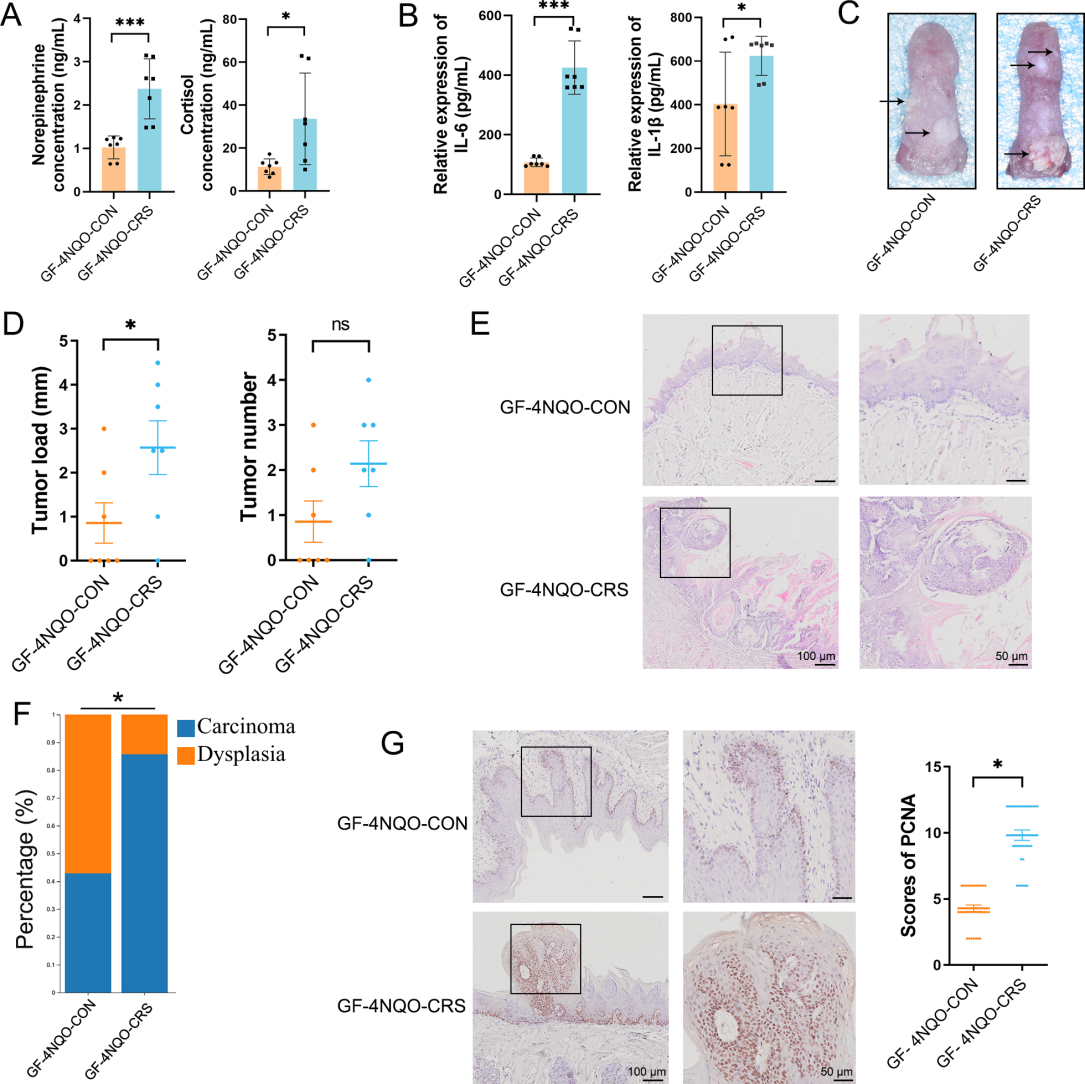
CRS-altered oral microbiota increases HNSCC tumourigenesis in GF mice. (**A**) Concentrations of NE and cortisol in GF mouse plasma determined by ELISA (GF-4NQO-CON group, n=7; GF-4NQO-CRS group, n=7). (**B**) Concentrations of IL-6 and IL-1β in GF mouse plasma determined by ELISA. (**C**) Representative images of tongue tissue at sacrifice. (**D**) Tumour size and tumour number in the GF-4NQO-CRS and GF-4NQO-CON groups. (**E**) Representative images of H&E staining showing dysplasia in the GF-4NQO-CON group and carcinoma in the GF-4NQO-CRS group. (**F**) Relative incidence of dysplasia and carcinoma in the tongue tissue from the GF-4NQO-CON group and the GF-4NQO-CRS group. Statistical significance was determined with the Fisher’s exact test. (**G**) Representative images of immunohistochemistry staining of PCNA-positive cells and the scores of PCNA-positive cells in the tongue tissue (n=5 fields from 7 mice). Data are expressed as mean±SD. Statistical significance was determined by two-tailed Student’s t-test. *p<0.05, ***p<0.001. CRS, chronic restraint stress; GF, germ-free; HNSCC, head and neck squamous cell carcinoma; NE, norepinephrine; 4NQO, 4-nitroquinoline-1-oxide; PCNA, proliferating cell nuclear antigen.

Similar to the conventional 4NQO mouse model, the secretion of NE and cortisol was significantly greater in the GF-4NQO-CRS group than in the GF-4NQO-CON group (figure 3A). Proinflammatory cytokines IL-6 and IL-1β were upregulated in the GF-4NQO-CRS group compared with the GF-4NQO-CON group (figure 3B). Bacterial dysbiosis is related to inflammation, which is associated with tumourigenesis.^15^ The GF-4NQO-CRS mice exhibited larger tumours than the GF-4NQO-CON mice, although there was no significant difference in tumour number (figure 3 C,D). Histological staining of tongue tissue confirmed increased prevalence of tumours in the GF-4NQO-CRS group than in the GF-4NQO-CON group (figure 3 E,F). Consistently, GF-4NQO-CRS mouse tongues showed greater cell proliferation than GF-4NQO-CON mouse tongues, which correlated with greater expression of PCNA (figure 3G). These results suggest that CRS-induced dysbiosis of the oral microbiota may directly promote HNSCC tumourigenesis in GF mice.

### Dysbiotic oral microbiota caused by CRS alters the host metabolome in GF mice

**Figure 4:**
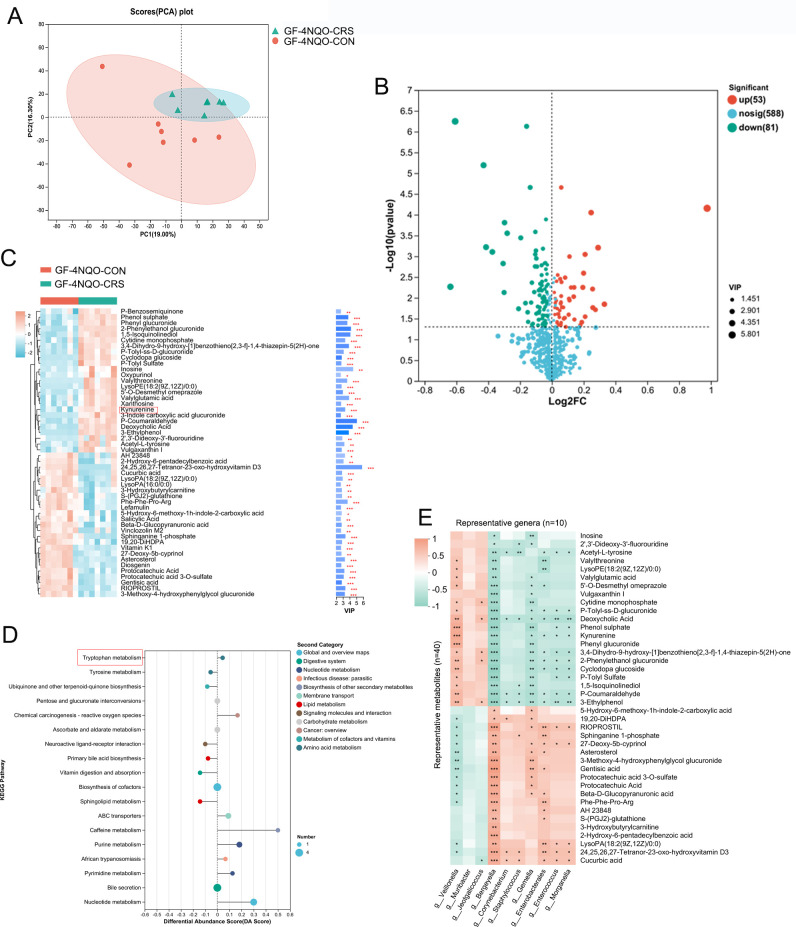
Dysbiotic oral microbiota caused by CRS alters the host metabolome in GF mice. (**A**) PCA analysis revealed a clear separation in the plasma metabolite compositions between the two groups of mice (GF-4NQO-CON group, n=7; GF-4NQO-CRS group, n=7). (**B**) Volcano plot of the change in metabolite concentration in GF-4NQO-CRS mouse plasma relative to that in GF-4NQO-CON mouse plasma. The x-axis denotes log_2_-transformed FC of metabolite abundances, and the y-axis indicates log_10_-transformed p values as determined by the Wilcoxon rank-sum test. The horizontal line represents a p value threshold of <0.05, and the vertical line represents FC thresholds of >1.5 or <0.67. Metabolites with concentrations that are elevated or decreased are highlighted in red or green, respectively. (**C**) Top 50 metabolites with different concentrations in the GF-4NQO-CRS and GF-4NQO-CON groups. VIP>2, p<0.05, two-tailed Mann-Whitney U test. (**D**) KEGG enrichment analysis of metabolites with different concentrations in the GF-4NQO-CRS and GF-4NQO-CON groups. (**E**) Heatmap depicting the relationships between the taxa and metabolites with different concentrations in the GF-4NQO-CRS and GF-4NQO-CON groups (Spearman analysis). *p<0.05, **p<0.01, ***p<0.001. CRS, chronic restraint stress; FC, fold change; GF, germ-free; KEGG, Kyoto encyclopaedia of genes and genomes; 4NQO, 4-nitroquinoline-1-oxide; PCA, principal component analysis; VIP, variable importance in projection.

Next, an untargeted metabolomic analysis was performed to identify the metabolic molecular signature in mouse plasma. Principal component analysis showed that there were significant differences in the plasma metabolite profiles of the GF-4NQO-CRS and GF-4NQO-CON groups (figure 4A), indicating a host metabolome shift associated with HNSCC tumourigenesis. Metabolites with significantly altered concentrations in the CRS group of mice, which may be important in the process of HNSCC tumourigenesis, were identified. Among these metabolites, 53 showed increased and 81 decreased abundance in the GF-4NQO-CRS mice compared with the GF-4NQO-CON mice (figure 4B). The top 50 metabolites with altered abundance included kynurenine (Kyn), indole, inosine, deoxycholic acid, phenyl glucuronide, cytidine monophosphate, acetyl-L-tyrosine, protocatechuic acid, fluorouridine, lysoPE(18:2(9Z,12Z)/0:0) and xanthosine, which were increased in the CRS mice, and hydroxyvitamin D_3_, vitamin K^1^, sphinganine 1-phosphate, 5-hydroxyindoleacetate, 3-hydroxyanthranilic acid and gentisic acid which were decreased in the CRS mice (figure 4C). Kyoto Encyclopaedia of Genes and Genomes (KEGG) enrichment analysis of the metabolome data identified 18 pathways that were modified between the GF- 4NQO-CRS and GF-4NQO-CON mice (figure 4D). Generally, these pathways belonged to nucleotide (purine and pyrimidine) metabolism, lipid metabolism (primary bile acid biosynthesis and sphingolipid), carbohydrate metabolism (pentose and glucuronate interconversions, ascorbate and aldarate), vitamin metabolism and amino acid metabolism (tryptophan and tyrosine). Among the amino acids, specifically, an increase in the concentrations of Kyn and 3-indole carboxylic acid glucuronide, along with a decrease in the levels of 5-hydroxyindoleacetate and 3-hydroxyanthranilic acid in the CRS mice, suggests that tryptophan (Trp) metabolism is affected by the CRS-altered oral microbiota. The Kyn metabolic pathway, which catabolises Trp, produces numerous bioactive metabolites.^16 17^

To further confirm the potential correlation between oral microbiota changes and changes in metabolite concentrations, Spearman correlation analysis was conducted on the altered bacteria and metabolite abundances. Overall, there was a significant correlation between the changes in bacterial genera and metabolite abundances (figure 4E). A significant positive correlation was observed between *Veillonella* and Kyn, while *Gemella*, *Enterococcus*, *Bergeyella* and *Morganella* were found to be depleted in GF-4NQO-CRS mice and exhibited a negative correlation with Kyn (figure 4E). *Enterococcus faecium* and *Morganella* supplements have been shown to affect tryptophan-Kyn metabolism.^18 19^ These results indicate that oral microbial dysbiosis induced by CRS and altered metabolite concentrations may work together to contribute to HNSCC tumourigenesis.

### CRS-induced oral microbial dysbiosis impairs mucosal barrier function in GF mice

**Figure 5:**
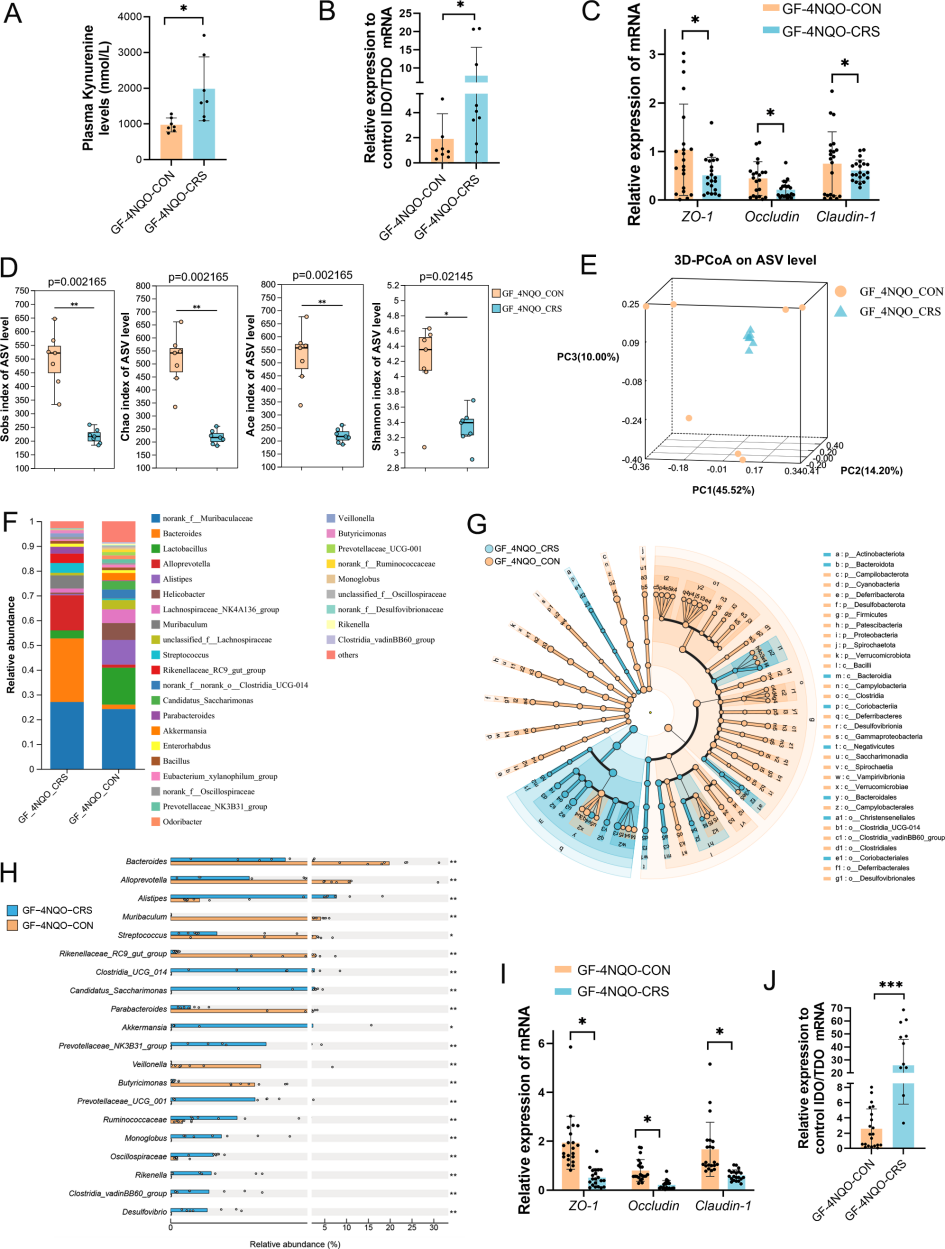
Altered oral microbiota by CRS impairs mucosal barrier function in GF mice. (**A**) UHPC-MS/MS quantification of the plasma Kyn concentration in GF-4NQO-CRS and GF-4NQO-CON mice (n=7). (**B**) qRT-PCR was used to calculate the ratio of IDO/TDO mRNA levels in the tongue tissues of GF-4NQO-CRS and GF-4NQO-CON mice. (**C**) qRT-PCR was used to measure the mRNA expression of *ZO-1*, *Occludin* and *Claudin-1* in the tongue tissues of GF-4NQO-CRS and GF-4NQO-CON mice. (**D**) Alpha-diversity indices (including Sobs, Chao1, Ace and Shannon indices) for faecal samples of GF-4NQO-CRS and GF-4NQO-CON mice. The significance of alpha diversity was assessed with a two-tailed Wilcoxon rank-sum test. (**E**) PCoA analysis (beta diversity) for faecal samples of GF-4NQO-CRS and GF-4NQO-CON mice. The significance of beta diversity was assessed by ANOSIM. (**F**) The composition of the microbiota at the genus level in faecal samples of GF-4NQO-CRS and GF-4NQO-CON mice. (**G**) Cladogram of the characterised microbes in each group, depicting their evolutionary and familial relationships. (**H**) Bacterial species from faecal samples that had the greatest change in abundance between the GF-4NQO-CRS group and the GF-4NQO-CON group and their relative abundance. (**I**) qRT-PCR was used to measure the mRNA expression of *ZO-1*, *Occludin* and *Claudin-1* in the colonic tissues of GF-4NQO-CRS and GF-4NQO-CON mice. (**J**) qRT-PCR was used to calculate the ratio of IDO/TDO mRNA levels in the colonic tissues of GF-4NQO-CRS and GF-4NQO-CON mice. Data are expressed as mean±SD. Statistical significance was determined with the two-tailed Student’s t-test. T. *p<0.05, **p<0.01, ***p<0.001. CRS, chronic restraint stress; GF, germ-free; IDO, indoleamine 2,3dioxygenase; 4NQO, 4-nitroquinoline-1-oxide; PCoA, principal coordinates analysis; qRT-PCR, quantitative reverse-transcription PCR; TDO, tryptophan 2,3dioxygenase; UHPC-MS/MS, ultrahigh performance liquid chromatography–tandem mass spectrometry.

UHPC-MS/MS was performed to further quantify the plasma Kyn concentrations. The results showed that the Kyn levels were significantly higher in the GF-4NQO-CRS group than in the GF-4NQO-CON group (figure 5A). Therefore, Kyn might be the metabolite associated with HNSCC tumourigenesis induced by CRS. Next, we investigated how the CRS-altered oral microbiota affected the plasma Kyn levels in GF mice. Indoleamine 2,3dioxygenase (IDO) and tryptophan 2,3dioxygenase (TDO) are the key enzymes in the degradation tryptophan into kynurenine.^20^ The IDO/TDO mRNA ratio was higher in the oral mucosal tissue of GF-4NQO-CRS mice than in GF-4NQO-CON mice (figure 5B), suggesting an increase in Kyn biosynthesis mediated by IDO/TDO. The integrity of oral mucosal barrier was measured by measuring ZO-1, Occludin, and Claudin-1 expression, and lower *ZO-1*, *Occludin*, and *Claudin-1* mRNA levels were observed in the GF-4NQO-CRS mice (figure 5C).

Kyn metabolism is closely related to the gut microbiota.^16 21^ 16S rRNA gene sequence analysis of faecal samples was used to identify alterations in the gut microbiota of GF-4NQO-CRS and GF-4NQO-CON mice. Alpha diversity (including Sobs, Chao1, Ace and Shannon indices) was significantly lower in the GF-4NQO-CRS mice than in the GF-4NQO-CON mice (figure 5D). 3D-PCoA based on Bray-Curtis distance revealed pronounced differentiation of the faecal microbiota for these two groups of mice (p<0.05, ANOSIM) (figure 5E). Community and LEfSe analysis also confirmed the distinct faecal microbial communities for the two groups (figure 5 F,G; online supplemental figure S2). Compared with the GF-4NQO-CON group, the abundance of some bacteria was significantly altered (p<0.05, FC>1.5) in the GF-4NQO-CRS group, and the top 20 altered genera are shown (figure 5H). These data indicated that CRS-altered oral microbiota could colonise the intestine and cause gut microbiota dysbiosis in GF mice with 4NQO treatment. Moreover, a significant reduction in the expression of *ZO-1*, *Occludin* and *Claudin-1* mRNA was observed in the colonic tissues of GF-4NQO-CRS mice relative to GF-4NQO-CON mice (figure 5I), suggesting that alteration in microbiota by CRS could impair gut barrier function. The IDO/TDO mRNA ratio also increased in the colon of GF-4NQO-CRS mice, which could lead to an overproduction of Kyn in the gut (figure 5J). Together, these data suggest that the CRS-altered oral microbiota may directly produce excessive Kyn within the oral cavity and gut, which can then enter the bloodstream through the damaged oral and gut mucosal barrier.

### Kynurenine in the chronic stress state promotes the progression of HNSCC by inhibiting aryl hydrocarbon receptor ubiquitination

**Figure 6:**
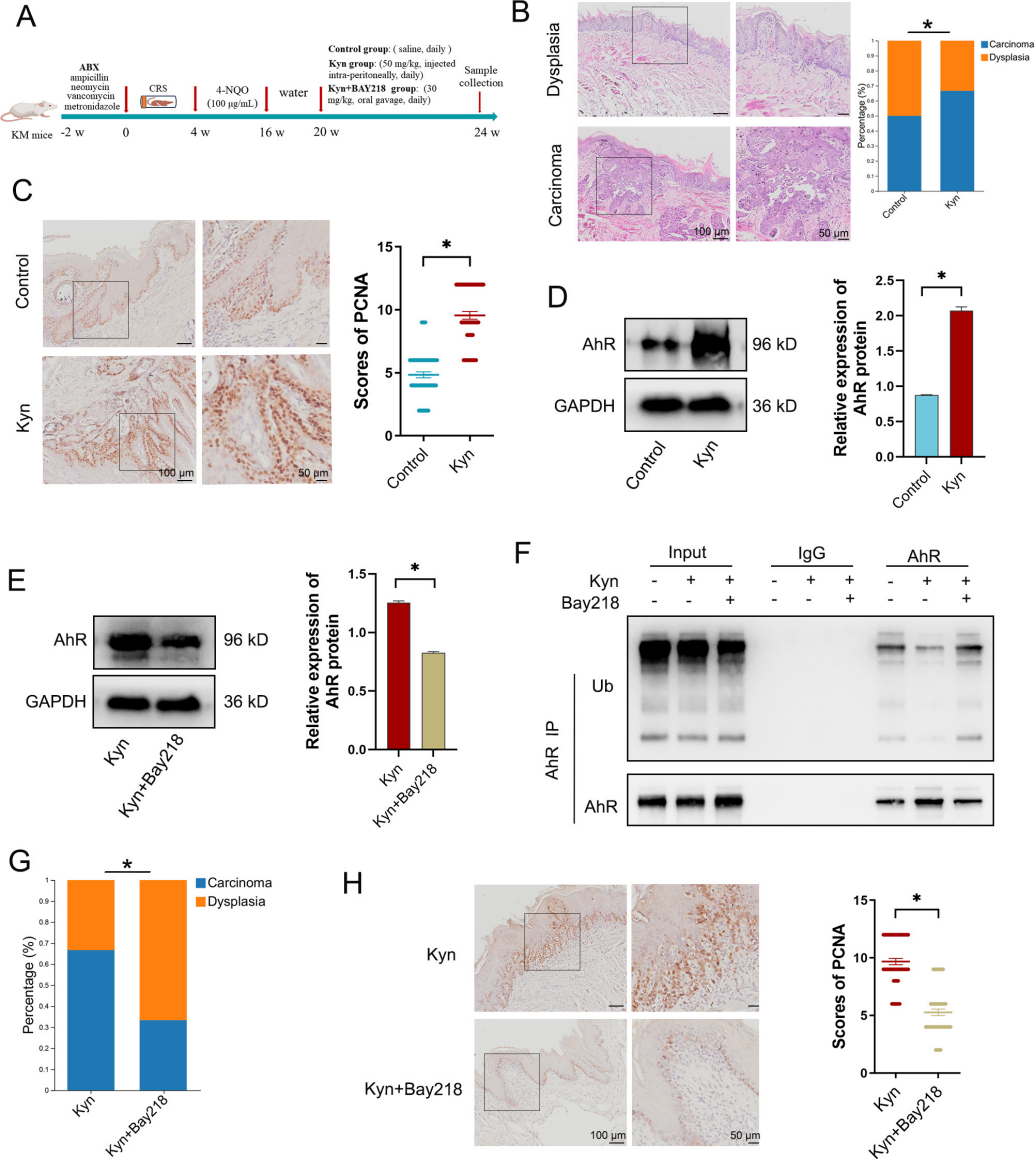
Kynurenine in the chronic stress state promotes the progression of HNSCC by inhibiting AhR ubiquitination. (**A**) Schematic overview of protocol: ABX-administered mice were subjected to the CRS treatment and 4NQO induction, with either separate or combined administration of Kyn and Bay218 (Control group, n=12; Kyn group, n=12; Kyn+Bay218 group, n=12). (**B**) Representative images of H&E staining of dysplasia in the Control group and carcinoma in the Kyn group. Prevalence of dysplasia and carcinoma in the tongue tissue in the Control group and Kyn group. Statistical significance was determined by Fisher’s exact test. (**C**) Representative images of immunohistochemical staining of PCNA-positive cells and the proportion of PCNA-positive cells in the tongue tissue (n=5 fields from 12 mice). (**D, E**) NE-stimulated HN6 cells were treated with Kyn or a combination of Kyn and Bay218. The expression of AhR was determined by western blot. (**F**) NE-stimulated HN6 cells were treated with Kyn or a combination of Kyn and Bay218. The cell lysates were immunoprecipitated with an anti-AhR antibody and immunoblotted with anti-ubiquitination antibody. (**G**) Prevalence of dysplasia and carcinoma in the tongue tissue from the Kyn group and the Kyn+Bay218 group. Statistical significance was determined by Fisher’s exact test. (**H**) Representative images of immunohistochemical staining of PCNA-positive cells and the proportion of PCNA-positive cells in the tongue tissue. Data are expressed as mean±SD. Statistical significance was determined by using a two-tailed Student’s t-test or one-way analysis of ANOVA. *p<0.05. ABX, antibiotic cocktail; AhR, aryl hydrocarbon receptor; ANOVA, analysis of variance; CRS, chronic restraint stress; HNSCC, head and neck squamous cell carcinoma; Kyn, Kynurenine; NE, norepinephrine; 4NQO, 4-nitroquinoline-1-oxide; PCNA, proliferating cell nuclear antigen; Ub, ubiquitination.

We then examined whether the microbial metabolite Kyn under CRS conditions has a promoting effect on HNSCC. ABX-treated mice were subjected to a 4-week CRS protocol. Then, all the mice were exposed to 4NQO induction, with or without concurrent Kyn treatment (figure 6A). An increased prevalence of squamous cell carcinoma was observed in the Kyn group (figure 6B). Moreover, Kyn promoted expression of PCNA, indicating an increased proliferative capacity of the epithelial cells (figure 6C). The mechanism underlying the Kyn promotion of HNSCC progression was studied further. The aryl hydrocarbon receptor (AhR) is a cytoplasmic receptor and transcription factor that senses xenobiotics and metabolites.^22^ NE has been widely used to replicate the chronic stress effect in vitro.^5^ Therefore, HN6 cells conditioned with NE were treated with cycloheximide (CHX), a protein-synthesis inhibitor, in the presence or absence of Kyn. Western blot analysis showed that Kyn enhanced the expression of the AhR protein (figure 6D). Conversely, suppression of AhR activity by the AhR inhibitor BAY218 resulted in the downregulation of AhR protein expression in HN6 cells (figure 6E), suggesting that Kyn regulates the stability of AhR. The ubiquitin-proteasome pathway is a principal mechanism for endogenous protein degradation within cells.^23^ It is hypothesised that the stability of the AhR protein may be controlled by suppressing the ubiquitin-proteasome pathway. Indeed, IP results showed that Kyn inhibited AhR ubiquitination, while BAY218 reinstated the ubiquitination in HN6 cells (figure 6F).

Next, in an 4NQO-induced ABX+CRS mouse model, mice were exposed to Kyn treatment, with or without concurrent BAY218 treatment. The results showed that BAY218 reduced the incidence of tumours post Kyn treatment (figure 6G) and reduced the expression of PCNA (figure 6H). Together, our data indicate that Kyn promotes the progression of HNSCC in chronic stress conditions by enhancing AhR stability through inhibiting AhR ubiquitination.

### Kynurenine in the chronic stress state regulates tumour-reactive CD8^+^ T cell exhaustion by activating the AhR

**Figure 7:**
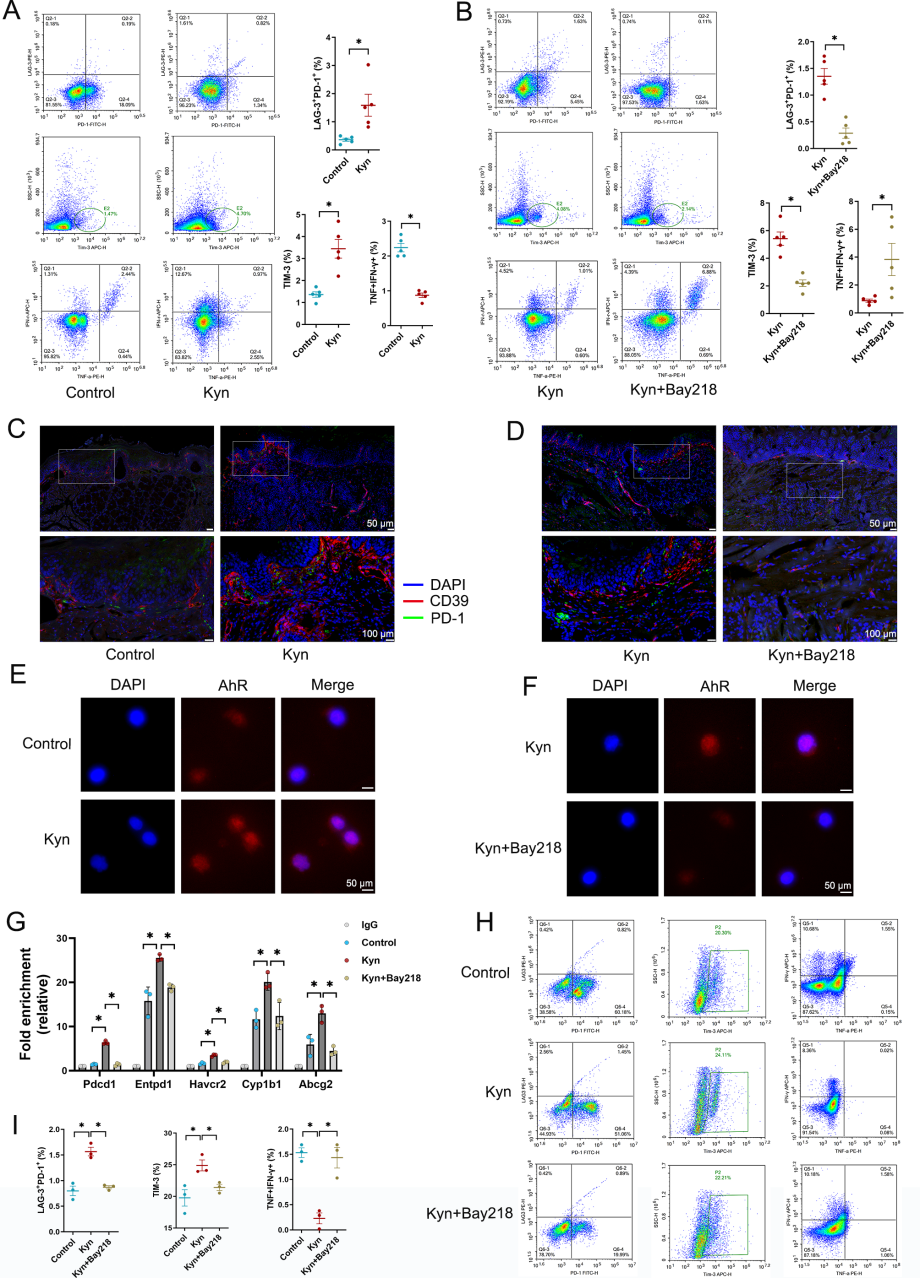
Kynurenine in the chronic stress state regulates tumour-reactive CD8^+^ T cell exhaustion by activating the AhR. (**A, B**) In the 4NQO-induced ABX+CRS mouse model, mice were administered Kyn with or without Bay218 (control=no Kyn or Bay218). CD8^+^ T cells were isolated from the spleens of the mice for flow cytometric analysis of the expression of IRs (PD-1, LAG-3, TIM-3) or of TNF and IFN-γ (n=5). (**C, D**) Representative images of mIHC staining of IRs in tongue tumour tissue; CD39 (red), PD-1 (green) and DAPI (blue). (**E, F**) NE-stimulated murine CD8^+^ T cells activated with anti-CD3/CD28 beads were treated with Kyn (**E**) or Kyn and Bay218 (**F**). T cells were stained for AhR. (**G**) NE-stimulated murine CD8^+^ T cells activated with anti-CD3/CD28 beads were treated with Kyn or Kyn+Bay218. ChIP–qPCR analysis was performed with AhR antibody and *Pdcd1*, *Entpd1*, *Havcr2*, *Cyp1b1* and *Abcg2* promotor-specific primers (n=3 independent experiments). (**H, I**) NE-stimulated murine CD8^+^ T cells activated with anti-CD3/CD28 beads were treated with Kyn or Kyn+Bay218. Flow cytometric analysis of the expression of IRs or of TNF and IFN-γ (n=3 independent experiments). Data are expressed as mean±SD. Statistical significance was determined using a two-tailed Student’s t-test or one-way analysis of ANOVA. *p<0.05. ABX, antibiotic cocktail; AhR, aryl hydrocarbon receptor; ANOVA, analysis of variance; CRS, chronic restraint stress; IRs, inhibitory receptors; Kyn, Kynurenine; NE, norepinephrine; 4NQO, 4-nitroquinoline-1-oxide.

Kyn, as a downstream metabolite of Trp metabolism, affects T cell proliferation and differentiation and mediates the immune evasion of tumour cells.^24 25^ To test whether Kyn inhibited antitumour immunity in HNSCC, we examined the effect of Kyn on the exhaustion of tumour-infiltrating lymphocytes in vivo. In a 4NQO-induced ABX+CRS mouse model, we found that Kyn treatment led to the exhaustion of CD8^+^ T cells derived from splenic lymphocytes, as evidenced by the increase expression of immune inhibitory receptors (IRs: programmed cell death receptor 1 (PD-1); lymphocyte activating 3 (LAG-3) and T-cell immunoglobulin and mucin-domain containing-3 (TIM-3)) and the reduced release of the effector cytokines interferon-γ (IFN-γ) and tumour necrosis factor (TNF) (figure 7A). However, the T cell exhaustion state caused by Kyn was effectively alleviated by BAY218 treatment of the mice (figure 7B). Kyn interacts with AhR, leading to the differentiation of T regulatory cells.^25^ The expression of CD8^+^ T cell markers was investigated in tumours from the 4NQO-induced ABX mouse model. Consistent with flow cytometry findings, the results of mIHC staining showed that Kyn treatment promoted the expression of PD-1 and CD39 (figure 7C), suggesting T cells entered an exhausted state. In addition, AhR inhibition with BAY218 reversed the T cell exhaustion caused by Kyn (figure 7D).

To explore the molecular pathway of the effect of Kyn on CD8^+^ T cell exhaustion under a chronic stress state, an in vitro assay was performed. In this assay, murine CD8^+^ T cells, stimulated by anti-CD3/CD28, were treated with Kyn, BAY218 and NE. Kyn promoted the nuclear translocation of AhR in CD8^+^ T cells stimulated with anti-CD3/CD28 antibody (figure 7E). The nuclear translocation of AhR was inhibited by BAY218 (figure 7F). ChIP-PCR confirmed that Kyn-activated AhR bound to the promoters of *Pdcd1*, *Entpd1*, *Havcr2*, *Cyp1b1* and *Abcg2*, while BAY218 inhibited this effect (figure 7G). Flow cytometry showed that Kyn increased the expression of IRs (PD-1, LAG-3, TIM-3) and reduced the levels of IFN-γ and TNF, while BAY218 had the opposite effect (figure 7 H,I). Together, our data suggest that, in the CRS state, Kyn triggers the exhaustion of CD8^+^ T cells by activating AhR and facilitates the progression of HNSCC.

## Discussion

CRS is a promoting factor for tumourigenesis.^26^ Even though several studies have demonstrated a connection between gut microbiota and CRS,^11 27^ it is still uncertain whether the alteration in oral microbiota caused by exposure to CRS could influence the initiation and progression of HNSCC. In this study, experiments were performed involving exposure of mice to CRS using the 4NQO-induced HNSCC mouse model and GF mice transplanted with CRS-altered oral microbiota. In addition, we explored the mechanism of CRS-induced microbially derived metabolite Kyn on the initiation and progression of HNSCC in vivo and in vitro.

Many studies have demonstrated that CRS enhances the growth of ovarian tumours in mice.^26 28^ CRS also promotes the growth and invasion of pancreatic and hepatocellular carcinoma by influencing the mobilisation and recruitment of myeloid-derived suppressor cells in the tumour microenvironment.^29^ A study has suggested, however, that CRS promotes lung metastasis of breast cancer rather than tumour growth.^3^ Researchers have obtained conflicting results while exploring the impact of CRS on various cancers, which may be attributed to the different responses of different tumours and cell lines to chronic stress. This study demonstrated that CRS could promote 4NQO-induced HNSCC tumourigenesis and reduce survival rate. However, until now the mechanism of CRS in HNSCC tumourigenesis was unknown.

CRS-induced neuroendocrine factors and inflammatory cytokines disrupt the gut–brain axis, leading to microbiome dysbiosis.^30^ The oral microbiome, the second largest microbial community in humans, plays a crucial role in the onset and progression of various diseases.^31^ Oral microbiome dysbiosis can alter the composition of the gut microbiome, promoting the progression of diseases.^13^ *Porphyromonas gingivalis* from the oral cavity compromises intestinal permeability and mediates immune responses associated with neurodegenerative diseases in mice.^32^ In this study, CRS promoted 4NQO-induced HNSCC tumourigenesis and induced oral microbiota dysbiosis. The oral microbiome dysbiosis caused by CRS was characterised by increased relative abundances of *Pseudomonas* and *Veillonella* as well as decreased abundances of some genera. *Pseudomonas* is a pathogen linked to widespread inflammation and infectious diseases. *Veillonellaceae* family members may contribute to the breakdown of epithelial barrier function by inducing the secretion of several proinflammatory mediators.^33^ Oral microbiota dysbiosis disrupts the host oral ecosystem homoeostasis and promotes the proliferation of pathogenic microorganisms, facilitating the progression to a chronic inflammatory state. Therefore, combined with the above literature and the results of this study, oral microbiota dysbiosis could be a key mediator in CRS-promoted HNSCC tumourigenesis. The direct promotional role of the dysbiotic oral microbiota induced by CRS in HNSCC tumourigenesis was confirmed in GF mice by oral microbiota transplantation.

KEGG enrichment analysis revealed that tryptophan (Trp) metabolism was the most significantly altered pathway in GF-4NQO-CRS mice. Host cells and microbes not only use Trp for protein synthesis but also use it to generate metabolites, through various metabolic pathways, which are involved in regulating host–microbiome interactions.^34^ Kyn, a key intermediate of Trp metabolism, plays a crucial role in regulating the immune system, central nervous system and tumour formation.^35 36^ In this study, our metabolomic analysis identified Kyn as the major carcinogenic metabolite in the plasma of GF-4NQO-CRS mice. Under conditions of elevated cortisol and inflammatory cytokine levels, the expression of TDO is suppressed while IDO is activated, promoting the conversion of Trp to Kyn.^37^ Consistent with previous findings,^20 21^ this study observed elevated levels of cortisol, IL-6 and IL-1β in the plasma, and increased IDO/TDO ratios in the oral mucosal and colonic tissues of GF-4NQO-CRS mice, indicating that the disrupted oral microbiome drives the biosynthesis of Trp towards Kyn. Certain bacteria exhibit either antitumour effects or promote carcinogenesis by producing metabolites.^38^ The regulatory interplay among microbiota, immune cells and epithelial cells is crucial for maintaining the structure and homeostasis of the mucosa.^39^ The mucosal epithelium recognises and responds to microbiota; conversely, microbial dysbiosis and consequential metabolite alterations disrupt the integrity and barrier function of the mucosal epithelium.^40^ This study found that CRS-induced oral microbiota changes caused impairment of the oral and intestinal barrier function in GF mice. Damaged tight junctions might enhance mucosal permeability, thereby permitting harmful metabolites to get into the oral cavity and intercellular space of epithelial cells.^15 41^

Given the close association between Kyn metabolism and inflammatory responses, Kyn is emerging as a critical player in various diseases including diabetes and cancer.^42^ A recent study has shown elevated Kyn levels and an increased plasma Kyn/Try ratio in patients with advanced breast cancer, which correlated with poor prognosis.^43^ The present study also found that CRS-induced oral microbiota leads to elevated plasma Kyn levels in GF mice which was associated with progression of HNSCC. Kyn acts as a potent immunomodulatory molecule, promoting immunosuppression during states of inflammation or infection and inducing the differentiation of regulatory T cells.^44^ In chronic infections and cancer, antigenic stimulation persists, preventing effective development of T cell memory and leading to T cell exhaustion.^45^ Both in vitro and in vivo evidence from this study confirmed that Kyn promotes the progression of HNSCC under chronic stress conditions and induces CD8^+^ T cell exhaustion. The mechanism by which Kyn interacts with CD8^+^ T cell exhaustion was investigated further. AhR is a cytoplasmic receptor and transcription factor activated by homologous ligand binding, and plays a critical role in immunity and tissue homeostasis.^22^ Kyn, an endogenous ligand for AhR, is involved in regulating the functional maturation of T cells.^22^ On binding with its ligand, AhR affects gene expression by binding to promoters, recruiting coactivators and corepressors to specific DNA regions, and interacting with signalling pathways.^46^ In this study, Kyn promoted CD8^+^ T cell exhaustion under stress conditions by stimulating the expression of the AhR target gene promoters for *Pdcd1*, *Entpd1*, *Havcr2*, *Cyp1b1* and *Abcg2*. In its inactive state, AhR resides in the cytoplasm and on activation binds with its nuclear transporter ARNT. The AhR-ARNT heterodimer enters the nucleus, initiating the transcription of target genes.^22 47^ This study further confirmed that under chronic stress conditions, Kyn promotes nuclear translocation of AhR in CD8^+^ T cells, indicating AhR activation. Moreover, Kyn inhibited the ubiquitination of AhR in CD8^+^ T cells. The ubiquitin-proteasome pathway is the primary method for endogenous protein degradation within cells, and the stability of the AhR protein is regulated by inhibiting this pathway.^48^ Interventions that affect AhR can impact the outcome of immune diseases, making AhR a potential target for immunotherapy.

Although the current study yields pertinent findings, it is essential to recognise its limitations. First, this study is based on a murine model and does not include human subjects, necessitating further investigation to determine the applicability of these findings to human physiology. Additionally, CRS models, while valuable, have limitations in capturing the complexity of the human stress experience. Therefore, the implications of our results for human stress physiology require further elucidation through more comprehensive and human-centric research methodologies.

In conclusion, this study demonstrated that CRS promotes HNSCC tumourigenesis by inducing oral microbiota dysbiosis, which affects metabolite levels, especially Kyn. Oral and gut barrier dysfunction caused by oral microbiota dysbiosis may further facilitate increased amounts of Kyn to enter the circulation. Moreover, under stress conditions, Kyn promotes CD8^+^ T cell exhaustion and HNSCC tumourigenesis through stabilising AhR by its deubiquitination. Our research indicates that the oral microbiota-Kyn-AhR pathway might serve as a novel therapeutic target for stress-induced HNSCC therapy.

## Supplementary material

10.1136/gutjnl-2024-333479 online supplemental file 1

10.1136/gutjnl-2024-333479 online supplemental file 2

## Data Availability

All data relevant to the study are included in the article or uploaded as online supplemental information.
