# Supplementary material for: Dysbiotic oral microbiota-derived kynurenine, induced by chronic restraint stress, promotes head and neck squamous cell carcinoma by enhancing CD8+ T cell exhaustion
Source: Gut. 2025 Feb 4;74(6):e333479. doi: 10.1136/gutjnl-2024-333479 (PMC12229062; doi:10.1136/gutjnl-2024-333479)
Supplement: online supplemental file 1 [file gutjnl-74-6-s001.pdf]

Supplementary Methods

Chronic restraint stress mouse model

The CRS mice were constrained daily and placed in a well-ventilated 50 mL conical tube for 2~5 h. Both the CRS and control groups were subjected to food and water deprivation during the restraint stress period. The restraint stress process lasted for 4 weeks. Behavioral tests, including the open-field test (OFT), tail suspension test (TST) and forced swim test (FST). The experimental mice were given a minimum of 1 h to acclimatize in a quiet test room before the initiation of behavioral tests. For the OFT, mice were individually placed at the center of the black open-field apparatus (45×45×45 cm), and the spatial exploration behaviors were recorded for 5 min using a tracking software system. The total distance traveled, and distance traveled in the center of the OFT were quantified to evaluate the anxiety-like behavior. For the TST, mice were restrained by tying a black string 2 cm from the tip of their tails. The mice then were suspended by the string in an inverted position, using a small metal hook, for 5 min. The time spent immobile was used to evaluate depression-like behavior. For the FST, mice were individually introduced into a glass cylinder measuring 30 cm in height and 15 cm in diameter. The cylinder contained 18 cm of water at 23±2 °C, and the mice were kept in this environment for 5 min. The time spent immobile was used to assess depression-like behavior. All behavioral tests were recorded using a video-tracking apparatus (SMART, Barcelona, Spain) and analyzed using EthoVision XT 13.0 software.

4NQO-induced HNSCC mouse model

Mice were treated with 4-nitroquinoline-1-oxide (4NQO, Sigma, USA) in the drinking water at a concentration of 100 μg/mL. Fresh drinking water containing 4NQO was supplied weekly. The mice were exposed to 14 weeks of treatment with 4NQO to induce HNSCC, followed by water alone. For the 4NQO+CRS group, prior to 4NQO administration, mice were exposed to 4 weeks of CRS. Subsequently, the mice that were successfully identified as CRS models were subjected to 14 weeks of treatment with 4NQO. Both CRS-exposed and CRS-free mice were sacrificed at the end of week 24. Swab samples of the oral microbiome were taken before mice were sacrificed. At

sacrifice, the tumor number was recorded and tumor load (the sum of the mean diameters of all tumors in each mouse; mean diameter = (major diameter + minor diameter)/2) was assessed.[1, 2] Tumor tissue and adjacent tongue tissues were collected for histopathology, and long-term survival was recorded.

Oral microbiota transplantation experiments

Oral microbiota transplantation was performed as previously described.[3, 4] GF mice were transplanted with 150 μL of the oral bacterial sample (~ 5×10^6 CFU/mouse in 2% methylcellulose) from conventional mice exposed to CRS, or from controls. Fresh oral microbiota was prepared daily for transplantation over a three-day period. The oral bacterial samples from the same group of donor mice were combined to ensure GF mice received similar inoculations. Oral swabs were used to collect the oral microbiota for transplantation. This involved brushing the mucosal surfaces within the mice's mouths, followed by suspension of the samples in a saline solution. After centrifuging the suspension at 2500 x g for 5 min, the oral bacteria were resuspended in 2% methylcellulose. The GF mice also received 4NQO at 50 ppm in the drinking water to induce neoplasia. After 12 weeks, 4NQO was withdrawn and replaced with water alone. Swab samples of the oral microbiome were taken at week 20 after inoculation, and then the GF mice were sacrificed. Tumor numbers were recorded and tumor loads were measured. Tumor tissue and adjacent tongue tissues were collected.

16S ribosomal RNA sequencing

The extraction of genomic DNA from oral swabs and stool samples was performed using the MoBio PowerSoil DNA Isolation Kit (Carlsbad, California, USA). DNA library preparation and 16S rRNA gene sequencing were performed by Majorbio Bio-pharm Technology Co. Ltd (Shanghai, China). The V3-V4 regions of 16S rRNA genes were amplified using universal primers (338F 5'- ACTCCTACGGGAGGCAGCAG-3' and 806R 5'- GGACTACHVGGGTWTCTAAT-3') that contained adapter and barcode sequences. High-throughput sequencing analysis of bacterial rRNA genes was performed using the Illumina NovaSeq 6000 platform. Negative controls for DNA extraction and PCR amplification were set up in the experiment. Reads were

demultiplexed per sample, filtered, and aligned to each of the five amplified regions based on primer sequences. The Short Multiple Regions Framework (SMURF) method was applied to combine read counts from the five regions into a coherent profiling result, solving a maximum likelihood problem. The GreenGenes database (version 2013) was used as a reference. [5] To reduce noise variation at very low abundance levels, samples with fewer than 1000 normalized reads (including negative controls) and species with relative abundances below 10^-4 were excluded from further analysis. To account for frequent contaminants, any species prevalent in more than 7.5% of negative DNA/PCR controls or more than 7.5% of empty paraffin controls was completely removed. [5, 6] The 16S rRNA gene sequence data from Illumina sequencing were quality-filtered and analyzed using QIIME2 software (version 2020.6.0). The paired-end reads were assembled based on the overlap relationships between them, resulting in optimized data following the quality control and assembly procedures. Subsequently, the optimized data were processed using the DADA2 method for denoising the data to obtain Amplicon Sequence Variant (ASV) representative sequences and abundance information. Based on the ASVs, a series of statistical or visual analyses were performed, including taxonomic classification, community diversity assessment, differential species analysis, and correlation analysis. ANOISM analysis was used to evaluate differences among the samples in species complexity. NCBI 16S microbial database (Sequence Read Archive, http://www.ncbi.nlm.nih.gov/Traces/sra) was used to classify taxonomy.

Untargeted metabolomics analysis

Metabolite extraction from plasma samples, untargeted LC-MS/MS analysis and data preprocessing and annotation were conducted by Majorbio Bio-pharm Technology Co. Ltd. The procedure was performed as previously described.[7, 8] For the extraction of metabolites, 100 μL plasma sample was mixed with 400 μL of extraction solvent (comprising an equal ratio of acetonitrile to methanol). The mixture was then subjected to ultrasonic agitation in an ice bath for 10 min and incubation at -40 °C for 1 h to promote protein precipitation. This was followed by centrifugation at 2500 x g for 15 min at 4 °C. The supernatant was analyzed as follows. Equal volumes of supernatant

from each sample were combined to create the quality control (QC) sample. LC-MS/MS analysis was performed using an UHPLC system (Agilent Technologies, Santa Clara, CA) with a UPLC BEH Amide column (1.7 μm 2.1*100 mm, Waters Corporation, Milford, MA) coupled to Q Exactive HFX mass spectrometer (Orbitrap MS, Thermo). The QE HFX mass spectrometer was used to acquire MS/MS spectra on an information-dependent basis (IDA). The raw data were transformed to the mzXML format using ProteoWizard and processed using the R package XCMS (version 3.2). Subsequently, the MS2 database (BiotreeDB) was used in metabolite annotation. The metabolomic data were analyzed using the MetaboAnalystR R package. Adjusted *p* values were computed by applying the Benjamini-Hochberg procedure to correct for the false discovery rate (FDR) from the initial *p* values. Metabolites exhibiting significant changes were identified using a set of criteria that included a variable importance in projection (VIP) >2 and an adjusted p value < 0.05.

UHPLC-MS/MS analysis

To quantify the Kynurenine (Kyn) concentration in the plasma of GF mice, ultrahigh performance liquid chromatography-tandem mass spectrometry (UHPC-MS/MS) analysis was conducted by Majorbio Bio-pharm Technology Co. Ltd. The procedures were as previously described.[8] Briefly, 100 μL of each plasma sample was used for the UHPLC-MS/MS analysis. The UHPLC separation was conducted using an EXIONLC System (SCIEX AB), which was outfitted with a Waters ACQUITY UPLC HSS T3 (1.8 μm 100 × 2.1 mm, Waters Corporation, Milford, MA). The auto-sampler was maintained at 4 °C, with an injection volume fixed at 10 μL. Assay development employed a SCIEX 6500 QTRAP + triple quadrupole mass spectrometer (Sciex), equipped with an IonDrive Turbo V electrospray ionization (ESI) interface. MRM data acquisition and analysis were conducted using SCIEX Analyst Work Station Software (Version 1.6.3) and Sciex MultiQuant™ 3.0.3, respectively. The final concentration (cF) in nmol/L was derived by multiplying the calculated concentration (cC) in nmol/L by the dilution factor (Dil). The metabolite concentration (CM, nmol/L) was determined by multiplying the final concentration (cF, nmol/L) by the sample experiment concentration factor (CF) and the final volume (VF, μL), then dividing by the sample

volume (VS, μL).

Histological analysis

Tongue tissues were fixed with 4% paraformaldehyde and embedded in paraffin, sectioned and stained with hematoxylin and eosin (HE) (Solarbio, Beijing, China) according to the manufacturer's instructions. The sections were scanned and observed using Pannoramic scanner (3DHISTECH, Germany).

Immunohistochemical analysis

Tongue tissues were fixed with 4% paraformaldehyde, embedded in paraffin, sectioned and stained with immunohistochemistry (IHC). IHC staining was performed using the Rabbit Enhanced Polymer test system (ZSGB-BIO, PV-9001, Beijing, China). The primary antibody was an anti-Proliferating cell nuclear antigen (PCNA) antibody (13110S, Cell Signaling Technology, Danvers, Massachusetts, USA) at 1:4,000 dilution. Bound antibody was visualized with DAB chromogen (ZSGB-BIO, ZLI-9018, Beijing, China). The sections were scanned using a Panoramic scanner (OLYMPUS OlyVIA 3.1, Olympus Corporation, Japan) and analyzed.

Multiplex immunohistochemical (mIHC) analysis

Tongue tissues were fixed with 4% paraformaldehyde, embedded in paraffin, and sectioned. The sections underwent antigen retrieval and were blocked with 5% bovine serum albumin (BSA). The tissue sections were incubated with anti-CD39 (ab300065, Abcam) and anti-PD-1 (ab52587, Abcam) antibodies at 4°C overnight, followed by incubation with Alexa Fluor 488 goat anti-mouse IgG secondary antibody and CY3 labeled goat anti-rabbit IgG secondary antibody for 1 h at room temperature. The cell nuclei were stained with DAPI. The multispectral images were acquired using a Case Viewer system (3DHISTECH, Germany).

Enzyme linked immunosorbent assay (ELISA) analysis

The levels of norepinephrine (NE), cortisol, IL-6, and IL-1β in mouse plasma were measured using ELISA kits (Jianglai company, Shanghai, China) according to the manufacturer's instructions. The absorbance values were measured at 450 nm using a microplate reader (SpectraMAX iD5, USA) and sample concentration was calculated.

Cell lines and culture

The human HNSCC cell line HN6 was obtained from ATCC (ATCC, Manassas, Virginia, USA). The HN6 cells were cultured in Dulbecco's modified Eagle's medium (Gibco, Carlsbad, California, USA) supplemented with 10% fetal bovine plasma (Invitrogen Life Technology, Carlsbad, California, USA) and 1% penicillin-streptomycin (Gibco, Carlsbad, California, USA) at 37°C with 5% CO_2. HN6 cells conditioned with NE (10 μM) were treated with cycloheximide (CHX) (2 μg/mL), a protein-synthesis inhibitor, in the presence or absence of Kyn (500 μM) and BAY218 (1 μM). After 48 h, cells were collected for analysis.

Quantitative reverse-transcription PCR (qRT-PCR)

Total RNA was extracted from mouse colon or oral mucous tissues with Trizol Reagent (Beyotime, China). Reverse transcription was conducted using a PrimeScript RT Reagent Kit with gDNA Eraser (Takara, Japan). The qRT-PCR was performed using TB Green® Premix DimerEraser™ (Takara, Japan) with the QuantStudio™ 7 Flex Real-Time PCR System (Thermo Fisher Scientific). The sequences of primers used for qRT-PCR are shown in Table 1. The relative mRNA expression was calculated based on the $2^{-\Delta\Delta CT}$ method and normalized against GAPDH mRNA levels.

Table 1. The primer sequences used for qRT-PCR

Gene	Forward 5’-3’	Reverse 5’-3’
IDO	TCCGGTCACGAATGTGGAAC	AAGCTGCCCGTTCTCAATCA
TDO	GCAAACCTGTGTGGTCCTGA	AAACGGGCACCCACTCATAG
ZO-1	GCTGTGGGTAACTCCATCCT	GGCTGACAGTGGAAGTAGCA
Occludin	GGAGTTTCAGGTGAATGGGTCA	AAATGTCCAGGCTCCCAAGA
Claudin-1	CCCTTCAGCAGAGCAAGGTT	CCCTTCAGCAGAGCAAGGTT
GAPDH	GGTGAAGGTCGGTGTGAACG	CTCGCTCCTGGAAGATGGTG

Measuring the effect of Kyn on HNSCC under CRS conditions

To investigate the effects of the microbial metabolite Kyn on HNSCC under CRS conditions, all mice received an antibiotic cocktail (ABX) consisting of 1 g/L metronidazole (HY-B0318, MedChemExpress, Monmouth Junction, NJ, USA), 1 g/L

ampicillin (SY007, Beyotime, China), 1 g/L neomycin (ST2533, Beyotime, China), and 0.5 g/L vancomycin (ST2807, Beyotime, China) in their drinking water while being subjected to a 4-week chronic stress protocol. This stress induction treatment started 2 weeks before the establishment of the 4NQO-induced HNSCC.[9] Subsequently, these mice were randomly divided into the Kyn group (50 mg/kg, injected intraperitoneally, daily), the Kyn + BAY218 group (30 mg/kg, oral gavage, daily) and the Control group (same volume of saline, daily). These treatments were administered for a duration of 4 weeks until the end of the experiment when mice were euthanized, and tissues were collected.

Cell preparation and *in vitro* treatment

$CD8^+$ T cells were isolated from the peripheral blood of mice using a mouse $CD8^+$ T cell Isolation Kit (magnetic cell separation, Miltenyi Biotec). The isolated $CD8^+$ T cells were cultured in RPMI 1640 medium containing 10% FBS, 50 μM β-mercaptoethanol, 100 U/mL mrIL-2, and 1 μg/mL Concanavalin A and were activated with 2 μg/mL anti-CD3 antibody for 48 h. After activation, the $CD8^+$ T cells were treated with NE (10 μM), Kyn (500 μM), and BAY218 (1 μM) for 48 h. After the indicated time periods, cells were collected for analysis.

Immunofluorescent staining

$CD8^+$ T cells were fixed with 4% paraformaldehyde and permeabilized with 0.5% Triton X-100. The cells then were blocked with 5% BSA and incubated with anti-AhR antibodies (GTX637885, GeneTex) at 4°C overnight, followed by incubation with Alexa Fluor 594 goat anti-rabbit IgG secondary antibody for 1 h at room temperature. The cell nuclei were stained with DAPI and cells were mounted for confocal analysis.

Western blot analysis

The total protein content of cells was extracted by treatment with RIPA lysis buffer and protease inhibitor (Beyotime, China). Proteins were separated by SDS-PAGE and then electroblotted onto PVDF membranes (Merck Millipore, Ireland). Membranes were blocked with 5% BSA and incubated with primary antibodies at 4°C overnight. The following primary antibodies were used: rabbit anti-AhR (1:1000; GTX637885, GeneTex), anti-GAPDH (1:1000; 2118s, Cell Signaling Technology) and anti-ubiquitin

(1:1000; 58395s, Cell Signaling Technology). Then, the membranes were incubated with secondary antibody for 1 h. Protein blots were visualized using the ECL Plus ChemiDoc™ Touch MP system (BIO-RAD, WA, USA). GAPDH was used as the internal control.

Coimmunoprecipitation (Co-IP) assay

Immunoprecipitation experiments were performed using a Pierce™ Classic Magnetic IP/Co-IP Kit (88804, Thermo Fisher Scientific, MA, USA) according to the manufacturer's protocol. Briefly, cells were lysed in IP buffer, and 25 μL of Pierce Protein A/G Magnetic Beads was added to 1mg of total protein and incubated for 1 h at room temperature. The tubes were placed in a magnetic stand to collect the beads and the supernatant was removed. The collected magnetic beads were incubated with 2 μg of anti-AhR antibody (sc-133088, Santa Cruz, CA, USA) and anti-rabbit IgG (2729S, Cell Signaling Technology) for 2 h at room temperature. The precipitated protein complex was collected with a magnetic stand, and then the beads were washed with IP Lysis/Wash Buffer. The beads were added to 100 μL of Lane Marker Sample Buffer (diluted five-fold with deionized water) and the samples were heated for 10 min at 96-100°C. The beads were then magnetically separated, and the supernatant containing the target antigen was retained. Twenty micrograms of the total protein was used as an input control. Western blot analysis was performed on the samples anti-AhR and anti-ubiquitin antibodies, as described above.

Chromatin Immunoprecipitation followed by qPCR (ChIP-qPCR)

ChIP-qPCR was conducted by using a SimpleChIP Enzymatic Chromatin IP Kit (9002S, Cell Signaling Technology) according to the manufacturer's protocol. Briefly, the chromatin from cross-linked $CD8^+$ T cells was isolated and fragmented. Subsequently, cells were immunoprecipitated with anti-AhR antibody (GTX637885, GeneTex). Anti-rabbit IgG (2729S, Cell Signaling Technology) was used as control. The primer sequences used for ChIP-qPCR are shown in Table 2. The fold enrichment method was used to normalize chromatin immunoprecipitation data.

Table 2 The primer sequences used for ChIP-qPCR

Gene	Forward 5'-3'	Reverse 5'-3'
Pdcd1	TCCAGCACATGCACTGTTGA	TCACAGGGCCATTCAAAGCA
Entpd1	AAGACCAAGAGGCACAAACTGA	AGGTTTGTTCTGGGTCAGTCC
Havcr2	TGTGCTCAAGGGGAACTGAC	GCAAGAGCTCTGCCTTCGTA
Cyp1b1	ATCGGATGGCACACCACG	ACTATCGCACTCCCCTTCCT
Abcg2	AGGAATCACACCATCCAACAG	CTAGGAAGGCCGTTGTTGTTTC

Flow cytometry

Lymphocyte suspensions from spleen tissues were prepared using an animal spleen tissue lymphocyte isolation kit (TBD0019SOP, TBD, Tianjin, China). All samples were treated with Live/Dead dye (Zombie Aqua Fixable Viability Kit, Biolegend, China) for 15 min. Cells were then stained with APC anti-mouse CD366 (Tim-3) (119705, Biolegend), FITC anti-mouse CD279 (PD-1) (135213, Biolegend), and PE anti-mouse CD223 (LAG-3) (125207, Biolegend) antibodies. For intracellular cytokine staining, cells were incubated with PMA/Ionomycin mixture(1X) (CS1001, MULTI SCIENCES, China) and BFA/Monensin Mixture(1X) (CS1002, MULTI SCIENCES) for 4 h with shaking at 37 °C. Then, these cells were fixed and permeabilized by using a Fix & Perm Kit (GAS003/2, MULTI SCIENCES). Finally, cells were stained with PE anti-mouse TNF-α (506305, Biolegend), and APC anti-mouse IFN-γ (505809, Biolegend) antibody. The data were acquired and analyzed with a NovoCyte flow cytometer (ACEA) and FlowJo software (Tree Star, Ashland, USA).

References

1 Bai X, Wei H, Liu W*, et al.* Cigarette smoke promotes colorectal cancer through modulation of gut microbiota and related metabolites. *Gut* 2022;71:2439-50.

2 Zhao R, Coker OO, Wu J*, et al.* Aspirin Reduces Colorectal Tumor Development in Mice and Gut Microbes Reduce its Bioavailability and Chemopreventive Effects. *Gastroenterology* 2020;159:969-83 e4.

3 Xiao H, Fan Y, Li Y*, et al.* Oral microbiota transplantation fights against head and neck radiotherapy-induced oral mucositis in mice. *Computational and Structural*

Biotechnology Journal 2021;19:5898-910.

4 Stashenko P, Yost S, Choi Y*, et al.* The Oral Mouse Microbiome Promotes Tumorigenesis in Oral Squamous Cell Carcinoma. *mSystems* 2019;4.

5 Nejman D, Livyatan I, Fuks G*, et al*. The human tumor microbiome is composed of tumor type-specific intracellular bacteria. *Science*. 2020;368(6494):973-980.

6 Eisenhofer R, Minich JJ, Marotz C*, et al*. Contamination in Low Microbial Biomass Microbiome Studies: Issues and Recommendations. *Trends Microbiol*. 2019;27(2):105-117.

7 Coker OO, Liu C, Wu WKK*, et al.* Altered gut metabolites and microbiota interactions are implicated in colorectal carcinogenesis and can be non-invasive diagnostic biomarkers. *Microbiome* 2022;10.

8 Shi B, Zhang X, Song Z, et al. Targeting gut microbiota-derived kynurenine to predict and protect the remodeling of the pressure-overloaded young heart. *Sci Adv* 2023;9(28):eadg7417.

9 He Y, Fu L, Li Y*, et al.* Gut microbial metabolites facilitate anticancer therapy efficacy by modulating cytotoxic CD8(+) T cell immunity. *Cell Metab* 2021;33:988-1000 e7.

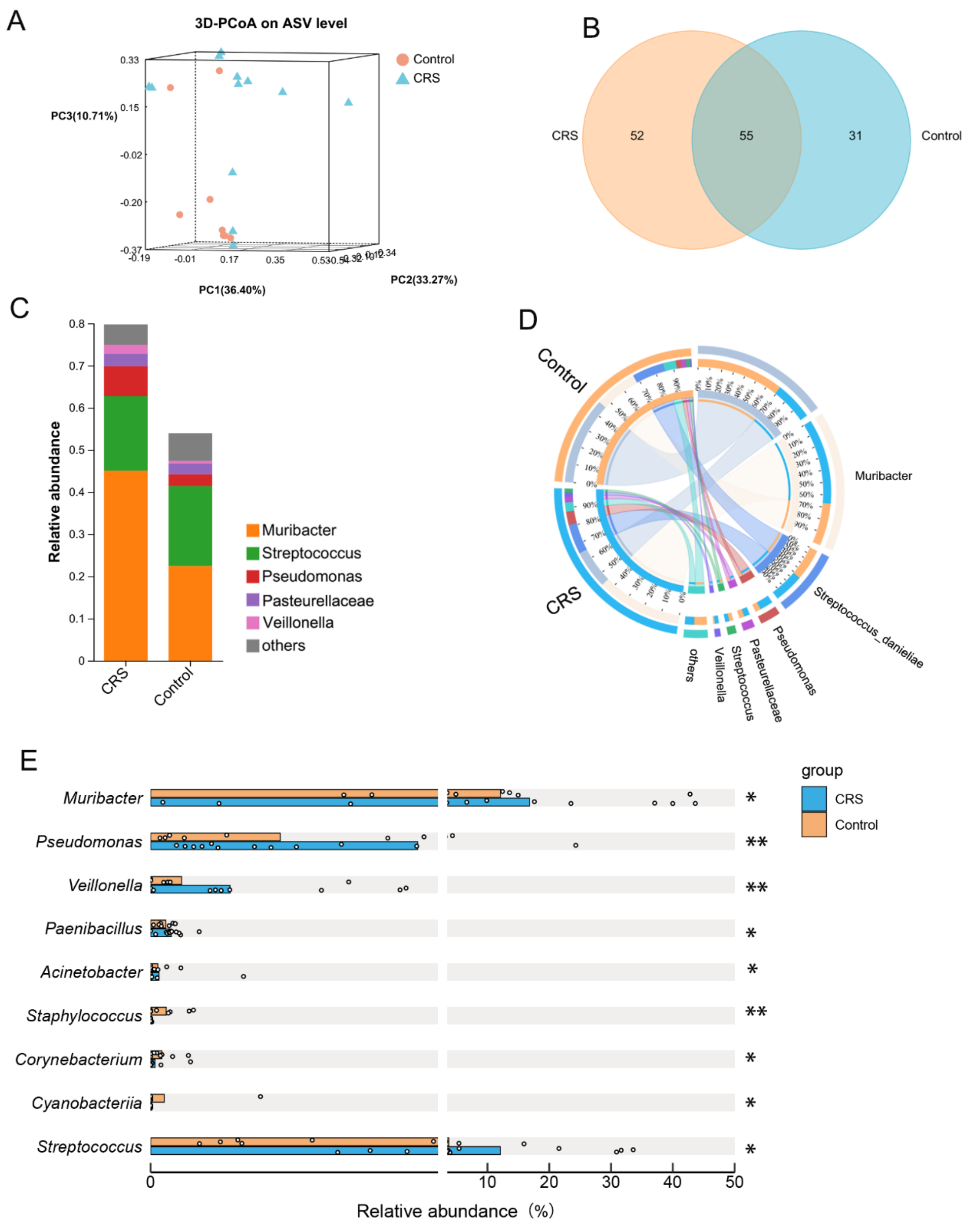

Figure S1 Chronic restraint stress alters the oral microbiota composition in mice. (A) PCoA analysis (beta diversity) at the ASV level of CRS and control mice (CRS group, n=12; Control group, n=8). **(B)** Venn plot showing the difference in bacterial genera between the two groups. **(C)** Composition of microbiota at the genus level in the CRS group and the control group. **(D)** Circos graph showed the distribution of microbial species at the genus level in the CRS group and control group. **(E)** Bacterial species from oral swabs that had the greatest change in abundance between the CRS

group and control group and their relative abundance. CRS, chronic restraint stress; PCoA, Principal coordinate analysis. *$p < 0.05$, **$p < 0.01$, ***$p < 0.001$.

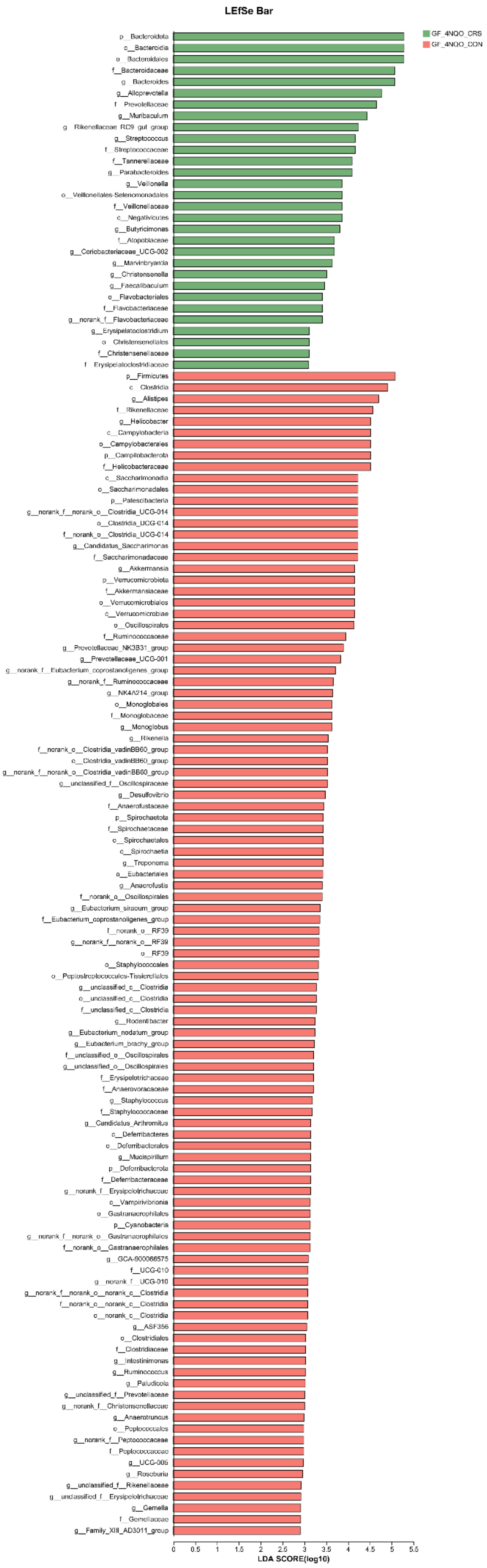
LEfSe Bar
GF_4NQO_CRS
GF_4NQO_CON
p__Bacteroidota
c__Bacteroidia
o__Bacteroidales
f__Bacteroidaceae
g__Bacteroides
g__Alloprevotella
f__Prevotellaceae
g__Muribaculum
g__Rikenellaceae_RC9_gut_group
g__Streptococcus
f__Streptococcaceae
f__Tannerellaceae
g__Parabacteroides
g__Veillonella
o__Veillonellales-Selenomonadales
f__Veillonellaceae
c__Negativicutes
g__Butyricimonas
f__Atopobiaceae
g__Coriobacteriaceae_UCG-002
g__Marvinbryantia
g__Christensenella
g__Faecalibaculum
o__Flavobacteriales
f__Flavobacteriaceae
g__norank_f__Flavobacteriaceae
g__Erysipelatoclostridium
o__Christensenellales
f__Christensenellaceae
f__Erysipelatoclostridiaceae
p__Firmicutes
c__Clostridia
g__Alistipes
f__Rikenellaceae
g__Helicobacter
c__Campylobacteria
o__Campylobacterales
p__Campilobacterota
f__Helicobacteraceae
c__Saccharimonadia
o__Saccharimonadales
p__Patescibacteria
g__norank_f__norank_o__Clostridia_UCG-014
o__Clostridia_UCG-014
f__norank_o__Clostridia_UCG-014
g__Candidatus_Saccharimonas
f__Saccharimonadaceae
g__Akkermansia
p__Verrucomicrobiota
f__Akkermansiaceae
o__Verrucomicrobiales
c__Verrucomicrobiae
o__Oscillospirales
f__Ruminococcaceae
g__Prevotellaceae_NK3B31_group
g__Prevotellaceae_UCG-001
g__norank_f__Eubacterium_coprostanoligenes_group
g__norank_f__Ruminococcaceae
g__NK4A214_group
o__Monoglobales
f__Monoglobaceae
g__Monoglobus
g__Rikenella
f__norank_o__Clostridia_vadinBB60_group
o__Clostridia_vadinBB60_group
g__norank_f__norank_o__Clostridia_vadinBB60_group
g__unclassified_f__Oscillospiraceae
g__Desulfovibrio
f__Anaerofustaceae
p__Spirochaetota
f__Spirochaetaceae
o__Spirochaetales
c__Spirochaetia
g__Treponema
o__Eubacteriales
g__Anaerofustis
f__norank_o__Oscillospirales
g__Eubacterium_siraeum_group
f__Eubacterium_coprostanoligenes_group
f__norank_o__RF39
g__norank_f__norank_o__RF39
o__RF39
o__Staphylococcales
o__Peptostreptococcales-Tissierellales
g__unclassified_c__Clostridia
o__unclassified_c__Clostridia
f__unclassified_c__Clostridia
g__Rodentibacter
g__Eubacterium_nodatum_group
g__Eubacterium_brachy_group
f__unclassified_o__Oscillospirales
g__unclassified_o__Oscillospirales
f__Erysipelotrichaceae
f__Anaerovoracaceae
g__Staphylococcus
f__Staphylococcaceae
g__Candidatus_Arthromitus
c__Deferribacteres
o__Deferribacterales
g__Mucispirillum
p__Deferribacterota
f__Deferribacteraceae
g__norank_f__Erysipelotrichaceae
c__Vampirivibrionia
o__Gastranaerophilales
p__Cyanobacteria
g__norank_f__norank_o__Gastranaerophilales
f__norank_o__Gastranaerophilales
g__GCA-900066575
f__UCG-010
g__norank_f__UCG-010
g__norank_f__norank_o__norank_c__Clostridia
f__norank_o__norank_c__Clostridia
o__norank_c__Clostridia
g__ASF356
o__Clostridiales
f__Clostridiaceae
g__Intestinimonas
g__Ruminococcus
g__Paludicola
g__unclassified_f__Prevotellaceae
g__norank_f__Christensenellaceae
g__Anaerotruncus
o__Peptococcales
g__norank_f__Peptococcaceae
f__Peptococcaceae
g__UCG-005
g__Roseburia
g__unclassified_f__Rikenellaceae
g__unclassified_f__Erysipelotrichaceae
g__Gemella
f__Gemellaceae
g__Family_XIII_AD3011_group
0
0.5
1
1.5
2
2.5
3
3.5
4
4.5
5
5.5
LDA SCORE(log10)

Figure S2 LEfSe showing the specific fecal microbes with greatest abundance that characterized each group of mice. LEfSe, linear discriminant analysis effect size.
