# Supplementary material for: Dysbiotic oral microbiota-derived kynurenine, induced by chronic restraint stress, promotes head and neck squamous cell carcinoma by enhancing CD8+ T cell exhaustion
Source: Gut. 2025 Feb 4;74(6):e333479. doi: 10.1136/gutjnl-2024-333479 (PMC12229062; doi:10.1136/gutjnl-2024-333479)
Supplement: online supplemental file 2 [file gutjnl-74-6-s002.pptx]

## Slide 1
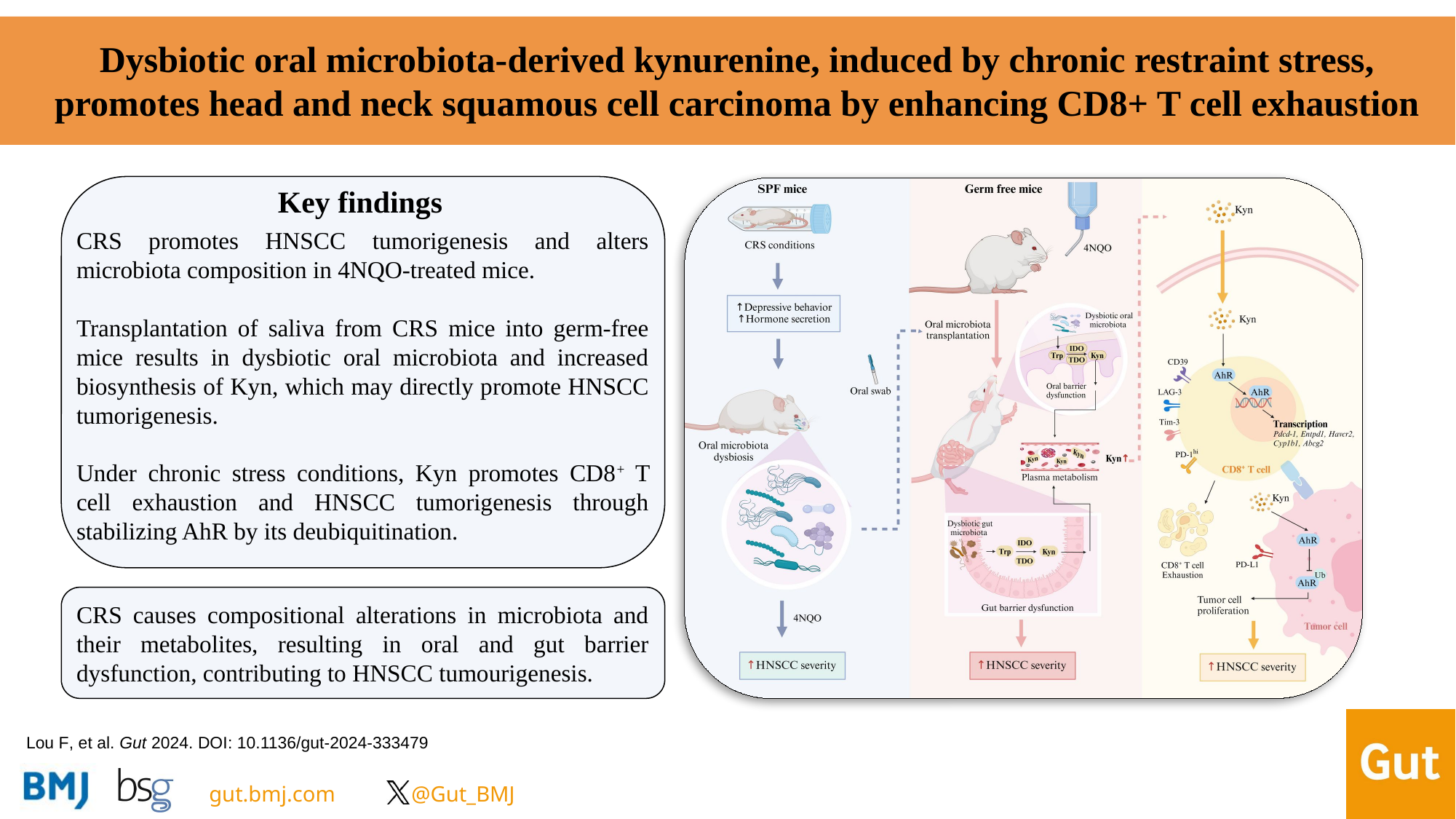

Dysbiotic oral microbiota-derived kynurenine, induced by chronic restraint stress, promotes head and neck squamous cell carcinoma by enhancing CD8+ T cell exhaustion
Key findings
CRS promotes HNSCC tumorigenesis and alters microbiota composition in 4NQO-treated mice.
Transplantation of saliva from CRS mice into germ-free mice results in dysbiotic oral microbiota and increased biosynthesis of Kyn, which may directly promote HNSCC tumorigenesis.
Under chronic stress conditions, Kyn promotes CD8+ T cell exhaustion and HNSCC tumorigenesis through stabilizing AhR by its deubiquitination.
CRS causes compositional alterations in microbiota and their metabolites, resulting in oral and gut barrier dysfunction, contributing to HNSCC tumourigenesis.
Lou F, et al. Gut 2024. DOI: 10.1136/gut-2024-333479
gut.bmj.com
@Gut_BMJ
